# Scaling deep learning for materials discovery

**DOI:** 10.1038/s41586-023-06735-9

**Published:** 2023-11-29

**Authors:** Amil Merchant, Simon Batzner, Samuel S. Schoenholz, Muratahan Aykol, Gowoon Cheon, Ekin Dogus Cubuk

**Affiliations:** 1 Google DeepMind, Mountain View, CA USA; 2 Google Research, Mountain View, CA USA

**Keywords:** Scaling laws, Computer science

## Abstract

Novel functional materials enable fundamental breakthroughs across technological applications from clean energy to information processing^1–11^. From microchips to batteries and photovoltaics, discovery of inorganic crystals has been bottlenecked by expensive trial-and-error approaches. Concurrently, deep-learning models for language, vision and biology have showcased emergent predictive capabilities with increasing data and computation^12–14^. Here we show that graph networks trained at scale can reach unprecedented levels of generalization, improving the efficiency of materials discovery by an order of magnitude. Building on 48,000 stable crystals identified in continuing studies^15–17^, improved efficiency enables the discovery of 2.2 million structures below the current convex hull, many of which escaped previous human chemical intuition. Our work represents an order-of-magnitude expansion in stable materials known to humanity. Stable discoveries that are on the final convex hull will be made available to screen for technological applications, as we demonstrate for layered materials and solid-electrolyte candidates. Of the stable structures, 736 have already been independently experimentally realized. The scale and diversity of hundreds of millions of first-principles calculations also unlock modelling capabilities for downstream applications, leading in particular to highly accurate and robust learned interatomic potentials that can be used in condensed-phase molecular-dynamics simulations and high-fidelity zero-shot prediction of ionic conductivity.

## Abstract (web summary)

A protocol using large-scale training of graph networks enables high-throughput discovery of novel stable structures and led to the identification of 2.2 million crystal structures, of which 381,000 are newly discovered stable materials.

## Main

The discovery of energetically favourable inorganic crystals is of fundamental scientific and technological interest in solid-state chemistry. Experimental approaches over the decades have catalogued 20,000 computationally stable structures (out of a total of 200,000 entries) in the Inorganic Crystal Structure Database (ICSD)^15,18^. However, this strategy is impractical to scale owing to costs, throughput and synthesis complications^19^. Instead, computational approaches championed by the Materials Project (MP)^16^, the Open Quantum Materials Database (OQMD)^17^, AFLOWLIB^20^ and NOMAD^21^ have used first-principles calculations based on density functional theory (DFT) as approximations of physical energies. Combining ab initio calculations with simple substitutions has allowed researchers to improve to 48,000 computationally stable materials according to our own recalculations^22–24^ (see Methods). Although data-driven methods that aid in further materials discovery have been pursued, thus far, machine-learning techniques have been ineffective in estimating stability (decomposition energy) with respect to the convex hull of energies from competing phases^25^.

In this paper, we scale up machine learning for materials exploration through large-scale active learning, yielding the first models that accurately predict stability and, therefore, can guide materials discovery. Our approach relies on two pillars: first, we establish methods for generating diverse candidate structures, including new symmetry-aware partial substitutions (SAPS) and random structure search^26^. Second, we use state-of-the art graph neural networks (GNNs) that improve modelling of material properties given structure or composition. In a series of rounds, these graph networks for materials exploration (GNoME) are trained on available data and used to filter candidate structures. The energy of the filtered candidates is computed using DFT, both verifying model predictions and serving as a data flywheel to train more robust models on larger datasets in the next round of active learning.

Through this iterative procedure, GNoME models have discovered more than 2.2 million structures stable with respect to previous work, in particular agglomerated datasets encompassing computational and experimental structures^15–17,27^. Given that discovered materials compete for stability, the updated convex hull consists of 381,000 new entries for a total of 421,000 stable crystals, representing an-order-of-magnitude expansion from all previous discoveries. Consistent with observations in other domains of machine learning^28^, we observe that our neural networks predictions improve as a power law with the amount of data. Final GNoME models accurately predict energies to 11 meV atom^−1^ and improve the precision of stable predictions (hit rate) to above 80% with structure and 33% per 100 trials with composition only, compared with 1% in previous work^17^. Moreover, these networks develop emergent out-of-distribution generalization. For example, GNoME enables accurate predictions of structures with 5+ unique elements (despite omission from training), providing one of the first strategies to efficiently explore this chemical space. We validate findings by comparing predictions with experiments and higher-fidelity r^2^SCAN (ref. ^29^) computations.

Finally, we demonstrate that the dataset produced in GNoME discovery unlocks new modelling capabilities for downstream applications. The structures and relaxation trajectories present a large and diverse dataset to enable training of learned, equivariant interatomic potentials^30,31^ with unprecedented accuracy and zero-shot generalization. We demonstrate the promise of these potentials for materials property prediction through the estimation of ionic conductivity from molecular-dynamics simulations.

## Overview of generation and filtration

The space of possible materials is far too large to sample in an unbiased manner. Without a reliable model to cheaply approximate the energy of candidates, researchers guided searches by restricting generation with chemical intuition, accomplished by substituting similar ions or enumerating prototypes^22^. Although improving search efficiency^17,27^, this strategy fundamentally limited how diverse candidates could be. By guiding searches with neural networks, we are able to use diversified methods for generating candidates and perform a broader exploration of crystal space without sacrificing efficiency.

**Fig. 1:**
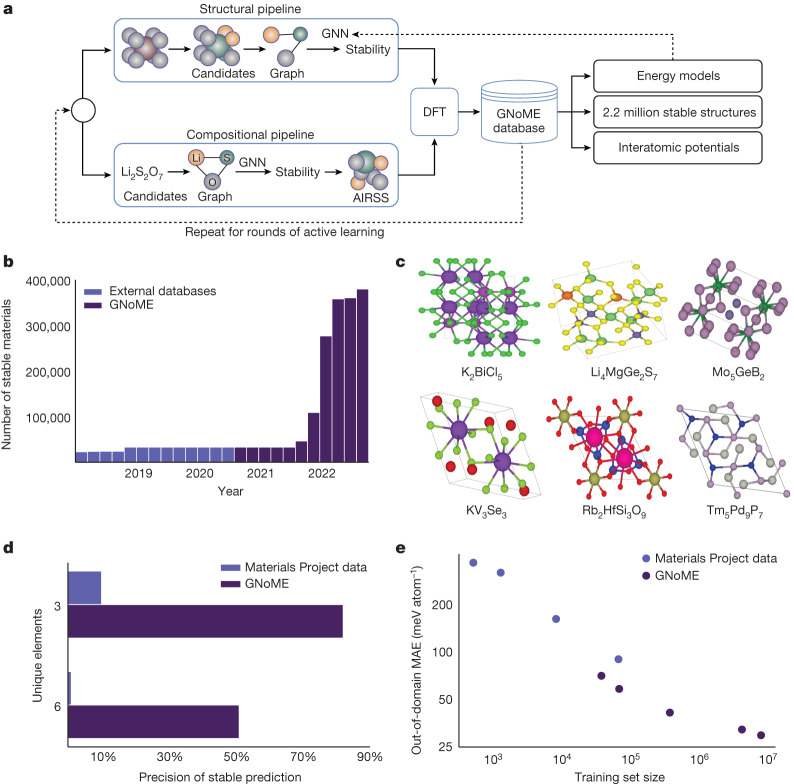
GNoME enables efficient discovery. **a**, A summary of the GNoME-based discovery shows how model-based filtration and DFT serve as a data flywheel to improve predictions. **b**, Exploration enabled by GNoME has led to 381,000 new stable materials, almost an order of magnitude larger than previous work. **c**, 736 structures have been independently experimentally verified, with six examples shown^50–55^. **d**, Improvements from graph network predictions enable efficient discovery in combinatorial regions of materials, for example, with six unique elements, even though the training set stopped at four unique elements. **e**, GNoME showcases emergent generalization when tested on out-of-domain inputs from random structure search, indicating progress towards a universal energy model.

To generate and filter candidates, we use two frameworks, which are visualized in Fig. 1a. First, structural candidates are generated by modifications of available crystals. However, we strongly augment the set of substitutions by adjusting ionic substitution probabilities to give priority to discovery and use newly proposed symmetry aware partial substitutions (SAPS) to efficiently enable incomplete replacements^32^. This expansion results in more than 10^9^ candidates over the course of active learning; the resulting structures are filtered by means of GNoME using volume-based test-time augmentation and uncertainty quantification through deep ensembles^33^. Finally, structures are clustered and polymorphs are ranked for evaluation with DFT (see Methods). In the second framework, compositional models predict stability without structural information. Inputs are reduced chemical formulas. Generation by means of oxidation-state balancing is often too strict (for example, neglecting Li_15_Si_4_). Using relaxed constraints (see Methods), we filter compositions using GNoME and initialize 100 random structures for evaluation through ab initio random structure searching (AIRSS)^26^. In both frameworks, models provide a prediction of energy and a threshold is chosen on the basis of the relative stability (decomposition energy) with respect to competing phases. Evaluation is performed through DFT computations in the Vienna Ab initio Simulation Package (VASP)^34^ and we measure both the number of stable materials discovered as well as the precision of predicted stable materials (hit rate) in comparison with the Materials Project^16^.

## GNoME

All GNoME models are GNNs that predict the total energy of a crystal. Inputs are converted to a graph through a one-hot embedding of the elements. We follow the message-passing formulation^35,36^, in which aggregate projections are shallow multilayer perceptrons (MLPs) with swish nonlinearities. For structural models, we find it important to normalize messages from edges to nodes by the average adjacency of atoms across the entire dataset. Initial models are trained on a snapshot of the Materials Project from 2018 of approximately 69,000 materials. Previous work benchmarked this task at a mean absolute error (MAE) of 28 meV atom^−1^ (ref. ^37^); however, we find that the improved networks achieve a MAE of 21 meV atom^−1^. We fix this promising architecture (see Methods) and focus on scaling in the rest of this paper.

### Active learning

A core step in our framework for accelerating materials discovery is active learning. In both structural and compositional frameworks, candidate structures filtered using GNoME are evaluated using DFT calculations with standardized settings from the Materials Project. Resulting energies of relaxed structures not only verify the stability of crystal structures but are also incorporated into the iterative active-learning workflow as further training data and structures for candidate generation. Whereas the hit rate for both structural and compositional frameworks start at less than 6% and 3%, respectively, performance improves steadily through six rounds of active learning. Final ensembles of GNoME models improve to a prediction error of 11 meV atom^−1^ on relaxed structures and hit rates of greater than 80% and 33%, respectively, clearly showing the benefits of scale. An analysis of final GNoME hit rates is provided in Fig. 1d.

### Scaling laws and generalization

The test loss performance of GNoME models exhibit improvement as a power law with further data. These results are in line with neural scaling laws in deep learning^28,38^ and suggest that further discovery efforts could continue to improve generalization. Emphatically, unlike the case of language or vision, in materials science, we can continue to generate data and discover stable crystals, which can be reused to continue scaling up the model. We also demonstrate emergent generalization to out-of-distribution tasks by testing structural models trained on data originating from substitutions on crystals arising from random search^26^ in Fig. 1e. These examples are often high-energy local minima and out of distribution compared with data generated by our structural pipeline (which, by virtue of substitutions, contains structures near their minima). Nonetheless, we observe clear improvement with scale. These results indicate that final GNoME models are a substantial step towards providing the community with a universal energy predictor, capable of handling diverse materials structures through deep learning.

## Discovered stable crystals

Using the described process of scaling deep learning for materials exploration, we increase the number of known stable crystals by almost an order of magnitude. In particular, GNoME models found 2.2 million crystal structures stable with respect to the Materials Project. Of these, 381,000 entries live on the updated convex hull as newly discovered materials.

**Fig. 2:**
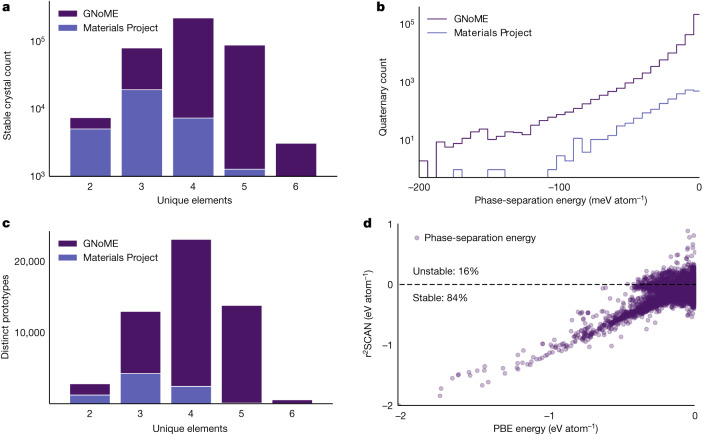
Summaries of discovered stable crystals. **a**, GNoME enables efficient discovery in the combinatorial spaces of 4+ unique elements that can be difficult for human experts. **b**, Phase-separation energies (energy to the convex hull) for discovered quaternaries showcase similar patterns but larger absolute numbers than previous catalogues. **c**, Discovered stable crystals correspond to 45,500 novel prototypes as measured by XtalFinder (ref. ^39^). **d**, Validation by r^2^SCAN shows that 84% of discovered binary and ternary crystals retain negative phase separations with more accurate functionals.

Consistent with other literature on structure prediction, the GNoME materials could be bumped off the convex hull by future discoveries, similar to how GNoME displaces at least 5,000 ‘stable’ materials from the Materials Project and the OQMD. See Supplementary Note 1 for discussion on improving structures of already-discovered compositions. Nevertheless, Figs. 1 and 2 provide a summary of the stable materials, with Fig. 1b focusing on the growth over time. We see substantial gains in the number of structures with more than four unique elements in Fig. 2a. This is particularly promising because these materials have proved difficult for previous discovery efforts^27^. Our scaled GNoME models overcome this obstacle and enable efficient discovery in combinatorially large regions.

Clustering by means of prototype analysis^39^ supports the diversity of discovered crystals with GNoME, leading to more than 45,500 novel prototypes in Fig. 2c (a 5.6 times increase from 8,000 of the Materials Project), which could not have arisen from full substitutions or prototype enumeration. Finally, in Fig. 2b, we compare the phase-separation energy (also referred to as the decomposition enthalpy) of discovered quaternaries with those from the Materials Project to measure the relative distance to the convex hull of all other competing phases. The similarities in distribution suggest that the found materials are meaningfully stable with respect to competing phases and not just ‘filling in the convex hull.’ Further analyses of materials near to (but not on) the updated convex hull is given in Supplementary Note 3.

### Validation through experimental matching and r^2^SCAN

All candidates for GNoME are derived from snapshots of databases made in March 2021, including the Materials Project and the OQMD. Concurrent to our discovery efforts, researchers have continued to experimentally create new crystals, providing a way to validate GNoME findings. Of the experimental structures aggregated in the ICSD, 736 match structures that were independently obtained through GNoME. Six of the experimentally matched structures are presented in Fig. 1c and further details of the experimental matches are provided in Supplementary Note 1. Similarly, of the 3,182 compositions added to the Materials Project since the snapshot, 2,202 are available in the GNoME database and 91% match on structure. A manual check of ‘newly’ discovered crystals supported the findings, with details in Supplementary Note 4.

We also validate predictions to ensure that model-based exploration did not overfit simulation parameters. We focus on the choice of functional. Standard projector augmented wave (PAW)-Perdew–Burke–Ernzerhof (PBE) potentials provided a speed–accuracy trade-off suited for large-scale discovery^40,41^, but the r^2^SCAN functional provides a more accurate meta-generalized gradient approximation^29,42,43^. 84% of the discovered binaries and ternary materials also present negative phase-separation energies (as visualized in Fig. 2d, comparable with a 90% ratio in the Materials Project but operating at a larger scale). 86.8% of tested quaternaries also remain stable on the r^2^SCAN convex hull. The discrepancies between PBE and r^2^SCAN energies are further analysed in Supplementary Note 2.

### Composition families of interest

We highlight the benefits of a catalogue of stable materials an order of magnitude larger than previous work. When searching for a material with certain desirable properties, researchers often filter such catalogues, as computational stability is often linked with experimental realizability. We perform similar analyses for three applications. First, layered materials are promising systems for electronics and energy storage^44^. Methods from previous studies^45^ suggest that approximately 1,000 layered materials are stable compared with the Materials Project, whereas this number increases to about 52,000 with GNoME-based discoveries. Similarly, following a holistic screening approach with filters such as exclusion of transition metals or by lithium fraction, we find 528 promising Li-ion conductors among GNoME discoveries, a 25 times increase compared with the original study^46^. Finally, Li/Mn transition-metal oxides are a promising family to replace LiCoO_2_ in rechargeable batteries^25^ and GNoME has discovered an extra 15 candidates stable relative to the Materials Project compared with the original nine.

## Scaling up learned interatomic potentials

The process of discovery of stable crystals also provides a data source beyond stable materials. In particular, the ionic relaxations involve computation of first-principles energies and forces for a diverse set of materials structures. This generates a dataset of unprecedented diversity and scale, which we explore to pretrain a general-purpose machine-learning interatomic potential (MLIP) for bulk solids. MLIPs have become a promising tool to accelerate the simulation of materials by learning the energies and forces of reference structures computed at first-principles accuracy^30,47–49^. Existing efforts typically train models per material, with data often sampled from ab initio molecular dynamics (AIMD). This markedly limits their general applicability and adoption, requiring expensive data collection and training a new potential from scratch for each system. By making use of the GNoME dataset of first-principles calculations from diverse structural relaxations, we demonstrate that large-scale pretraining of MLIPs enables models that show unprecedented zero-shot accuracy and can be used to discover superionic conductors, without training on any material-specific data.

### Zero-shot scaling and generalization

**Fig. 3:**
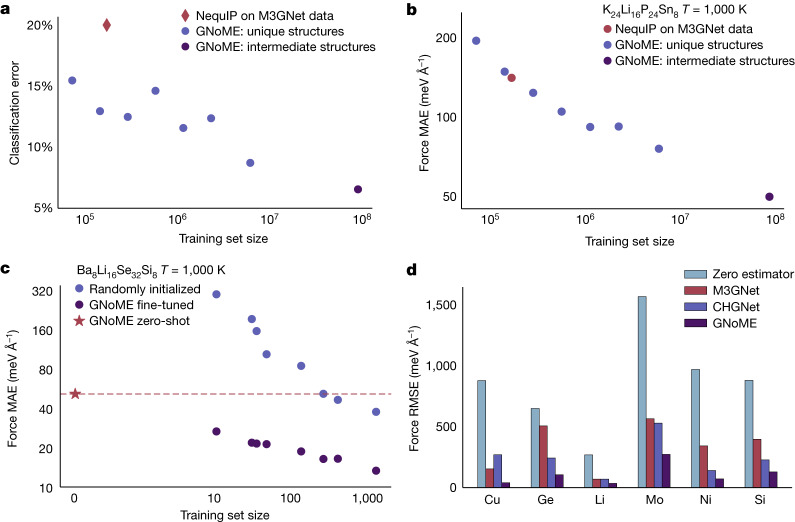
Scaling learned interatomic potentials. **a**, Classification of whether a material is a superionic conductor as predicted by GNoME-driven simulations in comparison with AIMD, tested on 623 unseen compositions. The classification error improves as a power law with training set size. **b**, Zero-shot force error as a function of training set size for the unseen material K_24_Li_16_P_24_Sn_8_. **c**, Robustness under distribution shift, showing the MAE in forces on the example material Ba_8_Li_16_Se_32_Si_8_. A GNoME-pretrained and a randomly initialized potential are trained on data of various sizes sampled at *T* = 400 K and evaluated on data sampled at *T* = 1,000 K. The zero-shot GNoME potential outperforms state-of-the-art models trained from scratch on hundreds of structures. **d**, Comparison of zero-shot force errors of three different pretrained, general-purpose potentials for bulk systems on the test set of ref. ^56^. Note that the composition Ni is not present in the GNoME pretraining data. RMSE, root-mean-square error.

We scale pretraining of a NequIP potential^30^ on data sampled from ionic relaxations. Increasing the pretraining dataset, we observe consistent power-law improvements in accuracy (see Fig. 3a,b). Despite only being trained on ionic relaxations and not on molecular-dynamics data, the pretrained GNoME potential shows remarkable accuracy when evaluated on downstream data sampled from the new distribution of AIMD in a zero-shot manner, that is, in which no training data originate from AIMD simulations (see Fig. 3). Notably, this includes unseen compositions, melted structures and structures including vacancies, all of which are not included in our training set (see Supplementary Note 6.4). In particular, we find that the scale of the GNoME dataset allows it to outperform existing general-purpose potentials (see Fig. 3d) and makes the pretrained potential competitive with models trained explicitly on hundreds of samples from the target data distributions (see Supplementary Note 6.4). We observe particularly pronounced improvements in the transferability of MLIPs, one of the most pressing shortcomings of MLIPs. To assess the transferability of the potentials, we test their performance under distribution shift: we train two types of NequIP potential on structures sampled from AIMD at *T* = 400 K, one in which the network is trained from randomly initialized weights and the other in which we fine-tune from a pretrained GNoME checkpoint. We then measure the performance of both potentials on data sampled from AIMD at *T* = 1,000 K (see Fig. 3c), out of distribution with respective to the 400-K data. The potential pretrained on GNoME data shows systematic and strong improvements in transferability over the potential trained from scratch, even when training is performed on more than 1,000 structures. The zero-shot GNoME potential, not fine-tuned on any data from this composition, outperforms even a state-of-the-art NequIP model trained on hundreds of structures.

### Screening solid-state ionic conductors

Solid electrolytes are a core component of solid-state batteries, promising higher energy density and safety than liquid electrolytes, but suffer from lower ionic conductivities at present. In the search for novel electrolyte materials, AIMD allows for the prediction of ionic conductivities from first principles. However, owing to the poor scaling of DFT with the number of electrons, routine simulations are limited to hundreds of picoseconds, hundreds of atoms and, most importantly, small compositional search spaces. Here we show that the GNOME potentials show high robustness in this out-of-distribution, zero-shot setting and generalizes to high temperatures, which allows them to serve as a tool for high-throughput discovery of novel solid-state electrolytes. We use GNoME potentials pretrained on datasets of increasing size in molecular-dynamics simulations on 623 never-before-seen compositions. Figure 3a shows the ability of the pretrained GNoME potentials to classify unseen compositions as superionic conductors in comparison with AIMD.

When scaled to the GNoME dataset—much larger than existing approaches—we find that deep learning unlocks previously impossible capabilities for building transferable interatomic potentials for inorganic bulk crystals and allows for high-accuracy, zero-shot prediction of materials properties at scale.

## Conclusion

We show that GNNs trained on a large and diverse set of first-principles calculations can enable the efficient discovery of inorganic materials, increasing the number of stable crystals by more than an order of magnitude. Associated datasets empower machine-learned interatomic potentials, giving accurate and robust molecular-dynamics simulations out of the box on unseen bulk materials. Our findings raise interesting questions about the capabilities of deep-learning systems in the natural sciences: the application of machine-learning methods for scientific discovery has traditionally suffered from the fundamental challenge that learning algorithms work under the assumption of identically distributed data at train and test times, but discovery is inherently an out-of-distribution effort. Our results on large-scale learning provide a potential step to move past this dilemma, by demonstrating that GNoME models exhibit emergent out-of-distribution capabilities at scale. This includes discovery in unseen chemical spaces (for example, with more than four different elements), as well as on new downstream tasks (for example, predicting kinetic properties).

GNoME models have already found 2.2 million stable crystals with respect to previous work and enabled previously impossible modelling capabilities for materials scientists. Some open problems remain for the transition of findings in applications, including a greater understanding of phase transitions through competing polymorphs, dynamic stability arising from vibrational profiles and configurational entropies and, ultimately, synthesizability. Nevertheless, we see pretrained, general-purpose GNoME models being used as powerful tools across a diverse range of applications to fundamentally accelerate materials discovery.

## Methods

### Datasets and candidate generation

#### Snapshots of available datasets

GNoME discoveries aim to extend the catalogues of known stable crystals. In particular, we build off previous work by the Materials Project^16^, the OQMD^17^, Wang, Botti and Marques (WBM)^27^ and the ICSD^15^. For reproducibility, GNoME-based discoveries use snapshots of the two datasets saved at a fixed point in time. We use the data from the Materials Project as of March 2021 and the OQMD as of June 2021. These structures are used as the basis for all discovery including via SAPS, yielding the catalogue of stable crystals as a result of GNoME. Further updates and incorporation of discoveries by these two groups could yield an even greater number of crystal discoveries.

For a revised comparison, another snapshot of the Materials Project, the OQMD and WBM was taken in July 2023. Approximately 216,000 DFT calculations were performed at consistent settings and used to compare the rate of GNoME discoveries versus the rate of discoveries by concurrent research efforts. From 2021 to 2023, the number of stable crystals external to GNoME expanded from 35,000 to 48,000, relatively small in comparison with the 381,000 new stable crystal structures available on the convex hull presented in this paper.

#### Substitution patterns

Structural substitution patterns are based on data-mined probabilities from ref. ^22^. That work introduced a probabilistic model for assessing the likelihood for ionic species substitution within a single crystal structure. In particular, the probability of substitution is calculated as a binary feature model such that $p(X,{X}^{{\prime} })\approx \frac{\exp {\sum }_{i}{\lambda }_{i}{f}_{i}^{(n)}(X,{X}^{{\prime} })}{Z}$, in which *X* and *X*′ are *n*-component vectors of *n* different ions. The model is simplified so that *f*_*i*_ is 0 or 1 if a specific substitution pair occurs and *λ*_*i*_ provides a weighting for the likelihood of a given substitution. The resulting probabilities have been helpful, for example, in discovering new quaternary ionic compounds with limited computation budgets.

In our work, we adjust the probabilistic model so as to increase the number of candidates and give priority to discovery. In particular, the conditional probability computation in the original substitution patterns prefers examples that are more likely to be found in the original dataset. For example, any uncommon element is assigned a smaller probability in the original model. To give priority to novel discovery and move further away from the known sets of stable crystals, we modify the implementation so that probabilities are only computed when two compositions differ. This minor modification has substantial benefits across our pipeline, especially when scaling up to six unique elements.

We also introduce changes to the model parameters to promote novel discovery. In the original probabilistic model, positive lambda refers to more likely substitutions, although ‘unseen’ or uncommon substitution resulted in negative lambda values. We increase the number of generations by setting the minimum value of any substitution pair to be 0. We then threshold high-probability substitutions to a value of 0.001, enabling efficient exploration in composition space through branch-and-bound algorithms available from pymatgen. Overall, these settings allow for many one-ion or two-ion substitutions to be considered by the graph networks that otherwise would not have been considered. We find this to be a good intermediate between the original model and using all possible ionic substitutions, in which we encounter combinatorial blow-ups in the number of candidates.

For the main part of this paper, substitutions are only allowed into compositions that do not match any available compositions in the Materials Project or in the OQMD, rather than comparing structures using heuristic structure matchers. This ensures that we introduce novel compositions in the dataset instead of similar structures that may be missed by structure matchers.

#### SAPS

To further increase the diversity of structures generations, we introduce a framework that we refer to as symmetry aware partial substitutions (SAPS), which generalizes common substitution frameworks. For a motivating example, consider the cases of (double) perovskites. Ionic substitutions on crystals of composition A_2_B_2_*X*_6_ does not lead to discovering double perovskites A_2_BB′O_6_, although the two only differ by a partial replacement on the B site.

SAPS enable efficient discovery of such structures. Starting with an original composition, we obtain candidate ion replacements using the probabilities as defined in the ‘Substitution patterns’ section. We then obtain Wyckoff positions of the input structures by means of symmetry analysers available through pymatgen. We enable partial replacements from 1 to all atoms of the candidate ion, for which at each level we only consider unique symmetry groupings to control the combinatorial growth. Early experiments limited the partial substitutions to materials that would charge-balance after partial substitutions when considering common oxidation states; however, greater expansion of candidates was achieved by removing such charge-balancing from the later experiments. This partial-substitution framework enables greater use of common crystal structures while allowing for the discovery of new prototypical structures, as discussed in the main part of this paper. Candidates from SAPS are from a different distribution to the candidates from full substitutions, which increases the diversity of our discoveries and our dataset.

To validate the impact of the SAPS, we traced reference structures from substitutions of all 381,000 novel stable structures back to a structure in the Materials Project or the OQMD by means of a topological sort (necessary as discovered materials were recycled for candidate generation). A total of 232,477 out of the 381,000 stable structures can be attributed to a SAPS substitution, suggesting notable benefit from this diverse candidate-generation procedure.

#### Oxidation-state relaxations

For the compositional pipeline, inputs for evaluation by machine-learning models must be unique stoichiometric ratios between elements. Enumerating the combinatorial number of reduced formulas was found to be too inefficient, but common strategies to reduce such as oxidation-state balancing was also too restrictive, for example, not allowing for the discovery of Li_15_Si_4_. In this paper, we introduce a relaxed constraint on oxidation-state balancing. We start with the common oxidation states from the Semiconducting Materials by Analogy and Chemical Theory (SMACT)^57^, with the inclusion of 0 for metallic forms. We allow for up to two elements to exist between two ordered oxidation states. Although this is a heuristic approach, it substantially improves the flexibility of composition generation around oxidation-state-balanced ratios.

#### AIRSS structure generation

Random structures are generated through AIRSS when needed for composition models^26^. Random structures are initialized as ‘sensible’ structures (obeying certain symmetry requirements) to a target volume and then relaxed through soft-sphere potentials. A substantial number of initializations and relaxations are needed to discover new materials, as different initial structures lead to different minima on the structure–energy landscape. For this paper, we always generate 100 AIRSS structures for every composition that is otherwise predicted to be within 50 meV of stable through composition-only model prediction.

As we describe in Supplementary Note 5, not all DFT relaxations converge for the 100 initializations per composition. In fact, for certain compositions, only a few initializations converge. One of the main difficulties arises from not knowing a good initial volume guess for the composition. We try a range of initial volumes ranging from 0.4 to 1.2 times a volume estimated by considering relevant atomic radii, finding that the DFT relaxation fails or does not converge for the whole range for each composition. Prospective analysis was not able to uncover why most AIRSS initializations fail for certain compositions, and future work is needed in this direction.

### Model training and evaluation

#### Graph networks

For structural models, edges are drawn in the graph when two atoms are closer than an interatomic distance cutoff (4.0 Å for structural models, 5.0 Å for interatomic potentials). Compositional models default to forming edges between all pairs of nodes in the graph. The models update latent node features through stages of message passing, in which neighbour information is collected through normalized sums over edges and representations are updated through shallow MLPs^36^. After several steps of message passing, a linear readout layer is applied to the global state to compute a prediction of the energy.

#### Training structural and composition models

Following Roost (representation learning from stoichiometry)^58^, we find GNNs to be effective at predicting the formation energy of a composition and structure.

For the structural models, the input is a crystal definition, which encodes the lattice, structure and atom definitions. Each atom is represented as a single node in the graph. Edges are defined when the interatomic distance is less than a user-defined threshold. Nodes are embedded by atom type, edges are embedded on the basis of the interatomic distance. We also include a global feature that is connected in the graph representation to all nodes. At every step of the GNN, neighbouring nodes and edge features are aggregated and used to update the corresponding representations of nodes, edges or globals individually. After 3–6 layers of message passing, an output layer projects the global vector to get an estimate of the energy. All data for training are shifted and scaled to approximately standardize the datasets. This structural model trained on the Materials Project data obtains state-of-the-art results of a mean absolute error of 21 meV atom^−1^. Training during the active-learning procedure leads to a model with a final mean absolute error of 11 meV atom^−1^. Training for structural models is performed with 1,000 epochs, with a learning rate of 5.55 × 10^−4^ and a linear decay learning rate schedule. By default, we train with a batch size of 256 and use swish nonlinearities in the MLP. To embed the edges, we use a Gaussian featurizer. The embedding dimension for all nodes and edges is 256 and, unless otherwise stated, the number of message-passing iterations is 3.

For the compositional models, the input composition to the GNN is encoded as a set of nodes, for which each element type in the composition is represented by a node. The ratio of the specific element is multiplied with the one-hot vector. For example, SiO_2_ would be represented with two nodes, in which one node feature is a vector of zeros and a 1/3 on the 14th row to represent silicon and the other node is a vector of zeros with a 2/3 on the 8th row to represent oxygen. Although this simplified GNN architecture is able to achieve state-of-the-art generalization on the Materials Project (MAE of 60 meV atom^−1^ (ref. ^25^)), it does not offer useful predictions for materials discovery, which was also observed by Bartel et al.^25^. One of the issues with compositional models is that they assume that the training label refers to the ground-state phase of a composition, which is not guaranteed for any dataset. Thus, the formation-energy labels in the training and test sets are inherently noisy, and reducing the test error does not necessarily imply that one is learning a better formation-energy predictor. To explore this, we created our own training set of compositional energies, by running AIRSS simulations on novel compositions. As described in Supplementary Note 5, we find that compositions for which there are only a few completed AIRSS runs tend to have large formation energies, often larger than predicted by the compositional GNN. We find that, if we limit ourselves to compositions for which at least ten AIRSS runs are completed, then the compositional GNN error is reduced to 40 meV atom^−1^. We then use the GNN trained on such a dataset (for which labels come from the minimum formation energy phase for compositions with at least ten completed AIRSS runs and ignoring the Materials Project data) and are able to increase the precision of stable prediction to 33%.

#### Model-based evaluation

Discovering new datasets aided by neural networks requires a careful balance between ensuring that the neural networks trained on the dataset are stable and promoting new discoveries. New structures and prototypes will be inherently out of distribution for models; however, we hope that the models are still capable of extrapolating and yielding reasonable predictions. This is out-of-distribution detection problem is further exacerbated by the implicit domain shift, in which models are trained on relaxed structures but evaluated on substitutions before relaxation. To counteract these effects, we make several adjustments to stabilize test-time predictions.

#### Test-time augmentations

Augmentations at test time are a common strategy for correcting instabilities in machine-learning predictions. Specific to structural models, we especially consider isotropic scaling of the lattice vectors, which both shrinks and stretches bonds. At 20 values ranging from 80% to 120% of the reference lattice scaling volume, we aggregate by means of minimum reduction. This has the added benefit of potentially correcting for predicting on nonrelaxed structures, as isotropic scaling may yield a more appropriate final structure.

#### Deep ensembles and uncertainty quantification

Although neural network models offer flexibility that allows them to achieve state-of-the-art performance on a wide range of problems, they may not generalize to data outside the training distribution. Using an ensemble of models is a simple, popular choice for providing predictive uncertainty and improving generalization of machine-learning predictions^33^. This technique simply requires training *n* models rather than one. The prediction corresponds to the mean over the outputs of all *n* models; the uncertainty can be measured by the spread of the *n* outputs. In our application of training machine-learning models for stability prediction, we use *n* = 10 graph networks. Moreover, owing to the instability of graph-network predictions, we find the median to be a more reliable predictor of performance and use the interquartile range to bound uncertainty.

#### Model-based filtration

We use test-time augmentation and deep-ensemble approaches discussed above to filter candidate materials based on energy. Materials are then compared with the available GNoME database to estimate the decomposition energy. Note that the structures provided for model-based filtration are unlikely to be completely related, so a threshold of 50 meV atom^−1^ was used for active learning to improve the recall of stable crystal discovery.

#### Clustered-based reduction

For active-learning setups, only the structure predicted to have the minimum energy within a composition is used for DFT verification. However, for an in-depth evaluation of a specific composition family of interest, we design clustering-based reduction strategies. In particular, we take the top 100 structures for any given composition and perform pairwise comparisons with pymatgen’s built-in structure matcher. We cluster the connected components on the graph of pairwise similarities and take the minimum energy structure as the cluster representation. This provides a scalable strategy to discovering polymorphs when applicable.

#### Active learning

Active learning was performed in stages of generation and later evaluation of filtered materials through DFT. In the first stage, materials from the snapshots of the Materials Project and the OQMD are used to generate candidates with an initial model trained on the Materials Project data, with a mean absolute error of 21 meV atom^−1^ in formation energy. Filtration and subsequent evaluation with DFT led to discovery rates between 3% and 10%, depending on the threshold used for discovery. After each round of active learning, new structural GNNs are trained to improve the predictive performance. Furthermore, stable crystal structures are added to the set of materials that can be substituted into, yielding a greater number of candidates to be filtered by the improved models. This procedure of retraining and evaluation was completed six times, yielding the total of 381,000 stable crystal discoveries. Continued exploration with active learning may continue to drive the number of stable crystals higher.

#### Composition-based hashing

Previous efforts to learn machine-learning models of energies often use a random split over different crystal structures to create the test set on which energy predictions are evaluated. However, as the GNoME dataset contains several crystal structures with the same composition, this metric is less trustworthy over GNoME. Having several structures within the same composition in both the training and the test sets markedly reduces test error, although the test error does not provide a measure of how well the model generalizes to new compositions. In this paper, we use a deterministic hash for the reduced formula of each composition and assign examples to the training (85%) and test (15%) sets. This ensures that there are no overlapping compositions in the training and test sets. We take a standard MD5 hash of the reduced formula, convert the hexadecimal output to an integer and take modulo 100 and threshold at 85.

### DFT evaluation

#### VASP calculations

We use the VASP (refs. ^34,59^) with the PBE^41^ functional and PAW^40,60^ potentials in all DFT calculations. Our DFT settings are consistent with the Materials Project workflows as encoded in pymatgen^23^ and atomate^61^. We use consistent settings with the Materials Project workflow, including the Hubbard *U* parameter applied to a subset of transition metals in DFT+U, 520 eV plane-wave-basis cutoff, magnetization settings and the choice of PBE pseudopotentials, except for Li, Na, Mg, Ge and Ga. For Li, Na, Mg, Ge and Ga, we use more recent versions of the respective potentials with the same number of valence electrons. For all structures, we use the standard protocol of two-stage relaxation of all geometric degrees of freedom, followed by a final static calculation, along with the custodian package^23^ to handle any VASP-related errors that arise and adjust appropriate simulations. For the choice of KPOINTS, we also force gamma-centred kpoint generation for hexagonal cells rather than the more traditional Monkhorst–Pack. We assume ferromagnetic spin initialization with finite magnetic moments, as preliminary attempts to incorporate different spin orderings showed computational costs that were prohibitive to sustain at the scale presented. In AIMD simulations, we turn off spin polarization and use the NVT ensemble with a 2-fs time step.

#### Bandgap calculations

For validation purposes (such as the filtration of Li-ion conductors), bandgaps are calculated for most of the stable materials discovered. We automate bandgap jobs in our computation pipelines by first copying all outputs from static calculations and using the pymatgen-based MPNonSCFSet in line mode to compute the bandgap and density of states of all materials. A full analysis of patterns in bandgaps of the novel discoveries is a promising avenue for future work.

#### r^2^SCAN

r^2^SCAN is an accurate and numerically efficient functional that has seen increasing adoption from the community for increasing the fidelity of computational DFT calculations. This functional is provided in the upgraded version of VASP6 and, for all corresponding calculations, we use the settings as detailed by MPScanRelaxSet and MPScanStaticSet in pymatgen. Notably, r^2^SCAN functionals require the use of PBE52 or PBE54 potentials, which can differ slightly from the PBE equivalents used elsewhere in this paper. To speed up computation, we perform three jobs for every SCAN-based computation. First, we precondition by means of the updated PBE54 potentials by running a standard relaxation job under MPRelaxSet settings. This preconditioning step greatly speeds up SCAN computations, which—on average—are five times slower and can otherwise crash on our infrastructure owing to elongated trajectories. Then, we relax with the r^2^SCAN functional, followed by a static computation.

### Metrics and analysis methodology

#### Decomposition energies

To compute decomposition energies and count the total number of stable crystals relative to previous work^16,17^ in a consistent fashion, we recalculated energies of all stable materials in the Materials Project and the OQMD with identical, updated DFT settings as enabled by pymatgen. Furthermore, to ensure fair comparison and that our discoveries are not affected by optimization failures in these high-throughput recalculations, we use the minimum energy of the Materials Project calculation and our recalculation when both are available.

#### Prototype analysis

We validate the novel discoveries using XtalFinder (ref. ^39^), using the compare_structures function available from the command line. This process was parallelized over 96 cores for improved performance. We also note that the symmetry calculations in the built-in library fail on less than ten of the stable materials discovered. We disable these filters but note that the low number of failures suggests minimal impact on the number of stable prototypes.

#### Families of interest

##### Layered materials

To count the number of layered materials, we use the methodology developed in ref. ^45^, which is made available through the pymatgen.analysis.dimensionality package with a default tolerance of 0.45 Å.

##### Li-ion conductors

The estimated number of viable Li-ion conductors reported in the main part of this paper is derived using the methodology in ref. ^46^ in a high-throughput fashion. This methodology involves applying filters based on bandgaps and stabilities against the cathode Li-metal anode to identify the most viable Li-ion conductors.

##### Li/Mn transition-metal oxide family

The Li/Mn transition-metal oxide family is discussed in ref. ^25^ to analyse the capabilities of machine-learning models for use in discovery. In the main text, we compare against the findings in the cited work suggesting limited discovery within this family through previous machine-learning methods.

#### Definition of experimental match

In the main part of this paper, we refer to experimentally validated crystal structures with the ICSD. More specifically, we queried the ICSD in January 2023 after many of crystal discoveries had been completed. We then extracted relevant journal (year) and chemical (structure) information from the provided files. By rounding to nearest integer formulas, we found 4,235 composition matches with materials discovered by GNoME. Of these, 4,180 are successfully parsed for structure. Then, we turn to the structural information provided by the ICSD. We used the CIF parser module of pymatgen to load the experimental ICSD structures into pymatgen and then compared those to the GNoME dataset using its structure matcher module. For both modules, we tried using the default settings as well as more tolerant settings that improve structure parsing and matching (higher occupancy tolerance in CIF parsing to fix cases with >1.0 total occupancy and allowing supercell and subset comparison in matching). The latter resulted in a slight increase (about 100) in the number of matched structures with respect to the default settings. Given that we are enforcing a strict compositional match, our matching process is still relatively conservative and is likely to yield a lower bound. Overall, we found 736 matches, providing experimental confirmation for the GNoME structures. 184 of these structures correspond to novel discoveries since the start of the project.

### Methods for creating figures of GNoME model scaling

Figures 1e and 3a,b show how the generalization abilities of GNoME models scale with training set size. In Fig. 1e, the training sets are sampled uniformly from the materials from the Materials Project and from our structural pipeline, which only includes elemental and partial substitutions into stable materials in the Materials Project and the OQMD. The training labels are the final formation energy at the end of relaxation. The test set is constructed by running AIRSS on 10,000 random compositions filtered by the SMACT. Test labels are the final formation energy at the end of the AIRSS relaxation, for crystals that AIRSS and DFT (both electronically and ionically) converged. Because we apply the same composition-based hash filtering (see ‘Composition-based hashing’ section) on all of our datasets, there is no risk of label leakage between the training set from the structural pipeline and the test set from AIRSS.

In Fig. 3a, we present the classification error for predicting the outcome of DFT-based molecular dynamics using GNN molecular dynamics. ‘GNoME: unique structures’ refers to the first step in the relaxation of crystals in the structural pipeline. We train on the forces on each atom on the first DFT step of relaxation. The different training subsets are created by randomly sampling compositions in the structural pipeline uniformly. ‘GNoME: intermediate structures’ includes all the same compositions as ‘GNoME: unique structures’, but has all steps of DFT relaxation instead of just the first step. The red diamond refers to the same GNN interatomic potential trained on the data from M3GNet, which includes three relaxation steps per composition (first, middle and last), as described in the M3GNet paper^62^.

### Coding frameworks

For efforts in machine learning, GNoME models make use of JAX and the capabilities to just-in-time compile programs onto devices such as graphics processing units (GPUs) and tensor processing units (TPUs). Graph networks implementations are based on the framework developed in Jraph, which makes use of a fundamental GraphsTuple object (encoding nodes and edges, along with sender and receiver information for message-passing steps). We also make great of use functionality written in JAX MD for processing crystal structures^63^, as well as TensorFlow for parallelized data input^64^.

Large-scale generation, evaluation and summarization pipelines make use of Apache Beam to distribute processing across a large number of workers and scale to the sizes as described in the main part of this paper (see ‘Overview of generation and filtration’ section). For example, billions of proposal structures, even efficiently encoded, requires terabytes of storage that would otherwise fail on single nodes.

Also, crystal visualizations are created using tooling from VESTA (ref. ^65^).

### MLIPs

#### Pretrained GNoME potential

We train a NequIP potential^30^, implemented in JAX using the e3nn-jax library^66^, with five layers, hidden features of 128 *ℓ* = 0 scalars, 64 *ℓ* = 1 vectors and 32 *ℓ* = 2 tensors (all even irreducible representations only, 128*x*0*e* + 64*x*1*x* + 32*x*2*e*), as well as an edge-irreducible representation of 0*e* + 1*e* + 2*e*. We use a radial cutoff of 5 Å and embed interatomic distances *r*_*ij*_ in a basis of eight Bessel functions, which is multiplied by the XPLOR cutoff function, as defined in HOOMD-blue (ref. ^67^), using an inner cutoff of 4.5 Å. We use a radial MLP *R*(*r*) with two hidden layers with 64 neurons and a SiLU nonlinearity. We also use SiLU for the gated, equivariant nonlinearities^68^. We embed the chemical species using a 94-element one-hot encoding and use a self-connection, as proposed in ref. ^30^. For internal normalization, we divide by 26 after each convolution. Models are trained with the Adam optimizer using a learning rate of 2 × 10^−3^ and a batch size of 32. Given that high-energy structures in the beginning of the trajectory are expected to be more diverse than later, low-energy structures, which are similar to one another and often come with small forces, each batch is made up of 16 structures sampled from the full set of all frames across all relaxations and 16 structures sampled from only the first step of the relaxation only. We found this oversampling of first-step structures to substantially improve performance on downstream tasks. The learning rate was decreased to a new value of 2 × 10^−4^ after approximately 23 million steps, to 5 × 10^−5^ after a further approximately 11 million steps and then trained for a final 2.43 million steps. Training was performed on four TPU v3 chips.

We train on formation energies instead of total energies. Formation energies and forces are not normalized for training but instead we predict the energy as a sum over scaled and shifted atomic energies, such that $\widehat{E}={\sum }_{i\in {N}_{{\rm{atoms}}}}\left({\widehat{{\epsilon }}}_{i}\sigma +\mu \right)$, in which ${\widehat{{\epsilon }}}_{i}$ is the final, scalar node feature on atom *i* and *σ* and *μ* are the standard deviation and mean of the per-atom energy computed over a single pass of the full dataset. The network was trained on a joint loss function consisting of a weighted sum of a Huber loss on energies and forces:

1 $${\mathcal{L}}={\lambda }_{E}\frac{1}{{N}_{{\rm{b}}}}\mathop{\sum }\limits_{b=1}^{b={N}_{{\rm{b}}}}{{\mathcal{L}}}_{{\rm{Huber}}}\left({\delta }_{E},\frac{{\widehat{E}}_{{\rm{b}}}}{{N}_{{\rm{a}}}},\frac{{E}_{{\rm{b}}}}{{N}_{{\rm{a}}}}\right)+{\lambda }_{F}\frac{1}{{N}_{{\rm{b}}}}\mathop{\sum }\limits_{b=1}^{b={N}_{{\rm{b}}}}\mathop{\sum }\limits_{a=1}^{b={N}_{{\rm{a}}}}{{\mathcal{L}}}_{{\rm{Huber}}}\left({\delta }_{F},-\frac{\partial \widehat{{E}_{{\rm{b}}}}}{\partial {r}_{{\rm{b}},{\rm{a}},\alpha }},{F}_{b,a,\alpha }\right)$$

in which *N*_a_ and *N*_b_ denote the number of atoms in a structure and the number of samples in a batch, respectively, ${\widehat{E}}_{{\rm{b}}}$ and *E*_b_ are the predicted and true energy for a given sample in a batch, respectively, and *F*_*a*,*α*_ is the true force component on atom *a*, for which *α* ∈ {*x*, *y*, *z*} is the spatial component. ${{\mathcal{L}}}_{{\rm{Huber}}}(\delta ,\widehat{a},a)$ denotes a Huber loss on quantity *a*, for which we use *δ*_*E*_ = *δ*_*F*_ = 0.01. The pretrained potential has 16.24 million parameters. Inference on an A100 GPU on a 50-atom system takes approximately 14 ms, enabling a throughput of approximately 12 ns day^−1^ at a 2-fs time step, making inference times highly competitive with other implementations of GNN interatomic potentials. Exploring new approaches with even further improved computational efficiency is the focus of future work.

#### Training on M3GNet data

To allow a fair comparison with the smaller M3GNet dataset used in ref. ^62^, a NequIP model was trained on the M3GNet dataset. We chose the hyperparameters in a way that balances accuracy and computational efficiency, resulting in a potential with efficient inference. We train in two setups, one splitting the training and testing sets based on unique materials and the other over all structures. In both cases, we found the NequIP potential to perform better than the M3GNet models trained with energies and forces (M3GNet-EF) reported in ref. ^62^. Given this improved performance, to enable a fair comparison of datasets and dataset sizes, we use the NequIP model trained on the structure-split M3GNet data in the scaling tests (the pretrained M3GNet model is used for zero-shot comparisons). We expect our scaling and zero-shot results to be applicable to a wide variety of modern deep-learning interatomic potentials.

The structural model used for downstream evaluation was trained using the Adam optimizer with a learning rate of 2 × 10^−3^ and a batch size of 16 for a total of 801 epochs. The learning rate was decreased to 2 × 10^−4^ after 601 epochs, after which we trained for another 200 epochs. We use the same joint loss function as in the GNoME pretraining, again with *λ*_*E*_ = 1.0, *λ*_*F*_ = 0.05 and *δ*_*E*_ = *δ*_*F*_ = 0.01. The network hyperparameters are identical to the NequIP model used in GNoME pretraining. To enable a comparison with ref. ^62^, we also subtract a linear compositional fit based on the training energies from the reference energies before training. Training was performed on a set of four V100 GPUs.

#### AIMD conductivity experiments

Following ref. ^69^, we classify a material as having superionic behaviour if the conductivity *σ* at the temperature of 1,000 K, as measured by AIMD, satisfies *σ*_1,000K_ > 101.18 mScm^−1^. Refer to the original paper for applicable calculations. See Supplementary Information for further details.

#### Robustness experiments

For the materials selected for testing the robustness of our models, As_24_Ca_24_Li_24_, Ba_8_Li_16_Se_32_Si_8_, K_24_Li_16_P_24_Sn_8_ and Li_32_S_24_Si_4_, a series of models is trained on increasing training set sizes sampled from the *T* = 400 K AIMD trajectory. We then evaluate these models on AIMD data sampled at both *T* = 400 K (to measure the effect of fine-tuning on data from the target distribution) and *T* = 1,000 K (to measure the robustness of the learned potentials). We trained two types of model: (1) a NequIP model from scratch and (2) a fine-tuned model that was pretrained on the GNoME dataset, starting from the checkpoint before the learning rate was reduced the first time. The network architecture is identical to that used in pretraining. Because the AIMD data contain fewer high-force/high-energy configurations, we use a L2 loss in the joint loss function instead of a Huber loss, again with *λ*_*E*_ = 1.0 and *λ*_*F*_ = 0.05. For all training set sizes and all materials, we scan learning rates 1 × 10^−2^ and 2 × 10^−3^ and batch sizes 1 and 16. Models are trained for a maximum of 1,000 epochs. The learning rate is reduced by a factor of 0.8 if the test error on a hold-out set did not improve for 50 epochs. We choose the best of these hyperparameters based on the performance of the final checkpoint on the 400-K test set. The 400-K test set is created using the final part of the AIMD trajectory. The training sets are created by sampling varying training set sizes from the initial part of the AIMD trajectory. The out-of-distribution robustness test is generated from the AIMD trajectory at 1,000 K. Training is performed on a single V100 GPU.

#### Molecular dynamics simulations

The materials for AIMD simulation are chosen on the basis of the following criteria: we select all materials in the GNoME database that are stable, contain one of the conducting species under consideration (Li, Mg, Ca, K, Na) and have a computationally predicted band gap >1 eV. The last criterion is chosen to not include materials with notable electronic conductivity, a desirable criterion in the search for electrolytes. Materials are run in their pristine structure, that is, without vacancies or stuffing. The AIMD simulations were performed using the VASP. The temperature is initialized at *T* = 300 K, ramped up over a time span of 5 ps to the target temperature, using velocity rescaling. This is followed by a 45-ps simulation equilibration using a Nosé–Hoover thermostat in the NVT ensemble. Simulations are performed at a 2-fs time step.

Machine-learning-driven molecular dynamics simulations using JAX MD^63^ are run on a subset of materials for which AIMD data were available and for which the composition was in the test set of the pretraining data (that is, previously unseen compositions), containing Li, Na, K, Mg and Ca as potentially conducting species. This results in 623 materials for which GNoME-driven molecular dynamics simulations are run. Simulations are performed at *T* =1,000 K using a Nosé–-Hoover thermostat, a temperature equilibration constant of 40 time steps, a 2-fs time step and a total simulation length of 50 ps. Molecular dynamics simulations are performed on a single P100 GPU.

For analysis of both the AIMD and the machine learning molecular dynamics simulation, the first 10 ps of the simulation are discarded for equilibration. From the final 40 ps, we compute the diffusivity using the DiffusionAnalyzer class of pymatgen with the default smoothed=max setting^23,70,71^.

## Online content

Any methods, additional references, Nature Portfolio reporting summaries, source data, extended data, supplementary information, acknowledgements, peer review information; details of author contributions and competing interests; and statements of data and code availability are available at 10.1038/s41586-023-06735-9.

### Supplementary information

Supplementary Information The supplementary information contains six sections, providing further context to the computational experiments performed.

## Data Availability

Crystal structures corresponding to stable discoveries discussed throughout the paper will be made available at https://github.com/google-deepmind/materials_discovery. In particular, we provide results for all stable structures, as well as any material that has been recomputed from previous datasets to ensure consistent settings. Associated data from the r^2^SCAN functional will be provided, expectantly serving as a foundation for analysing discrepancies between functional choices. Data will also be available via the Materials Project at https://materialsproject.org/gnome with permanent link: 10.17188/2009989.

## Code availability

Software to analyse stable crystals and associated phase diagrams, as well as the software implementation of the static GNN and the interatomic potentials, will be made available at https://github.com/google-deepmind/materials_discovery.

## References

[1] Green MA, Ho-Baillie A, Snaith HJ (2014). The emergence of perovskite solar cells. Nat. Photon..

[2] Mizushima K, Jones P, Wiseman P, Goodenough JB (1980). Li_*x*_CoO_2_ (0<*x*<-1): a new cathode material for batteries of high energy density. Mater. Res. Bull..

[3] Bednorz JG, Müller KA (1986). Possible high *T*_c_ superconductivity in the Ba–La–Cu–O system. Z. Phys. B Condens. Matter.

[4] Ceder G (1998). Identification of cathode materials for lithium batteries guided by first-principles calculations. Nature.

[5] Tabor DP (2018). Accelerating the discovery of materials for clean energy in the era of smart automation. Nat. Rev. Mater..

[6] Liu C (2020). Two-dimensional materials for next-generation computing technologies. Nat. Nanotechnol..

[7] Nørskov JK, Bligaard T, Rossmeisl J, Christensen CH (2009). Towards the computational design of solid catalysts. Nat. Chem..

[8] Greeley J, Jaramillo TF, Bonde J, Chorkendorff I, Nørskov JK (2006). Computational high-throughput screening of electrocatalytic materials for hydrogen evolution. Nat. Mater..

[9] Gómez-Bombarelli R (2016). Design of efficient molecular organic light-emitting diodes by a high-throughput virtual screening and experimental approach. Nat. Mater..

[10] de Leon NP (2021). Materials challenges and opportunities for quantum computing hardware. Science.

[11] Wedig A (2016). Nanoscale cation motion in TaO_*x*_, HfO_*x*_ and TiO_*x*_ memristive systems. Nat. Nanotechnol..

[12] Brown T (2020). Language models are few-shot learners. Adv. Neural Inf. Process. Syst..

[13] Dosovitskiy, A. et al. An image is worth 16x16 words: Transformers for image recognition at scale. In *International Conference on Learning Representations* (ICLR, 2021); https://openreview.net/forum?id=YicbFdNTTy

[14] Jumper J (2021). Highly accurate protein structure prediction with AlphaFold. Nature.

[15] Hellenbrandt M (2004). The Inorganic Crystal Structure Database (ICSD)—present and future. Crystallogr. Rev..

[16] Jain A (2013). Commentary: The Materials Project: a materials genome approach to accelerating materials innovation. APL Mater..

[17] Saal JE, Kirklin S, Aykol M, Meredig B, Wolverton C (2013). Materials design and discovery with high-throughput density functional theory: the Open Quantum Materials Database (OQMD). JOM.

[18] Belsky A, Hellenbrandt M, Karen VL, Luksch P (2002). New developments in the Inorganic Crystal Structure Database (ICSD): accessibility in support of materials research and design. Acta Crystallogr. B Struct. Sci..

[19] Aykol M, Montoya JH, Hummelshøj J (2021). Rational solid-state synthesis routes for inorganic materials. J. Am. Chem. Soc..

[20] Curtarolo S (2012). AFLOWLIB.ORG: a distributed materials properties repository from high-throughput ab initio calculations. Comput. Mater. Sci..

[21] Draxl C, Scheffler M (2019). The NOMAD laboratory: from data sharing to artificial intelligence. J. Phys. Mater..

[22] Hautier G, Fischer C, Ehrlacher V, Jain A, Ceder G (2011). Data mined ionic substitutions for the discovery of new compounds. Inorg. Chem..

[23] Ong SP (2013). Python Materials Genomics (pymatgen): a robust, open-source Python library for materials analysis. Comput. Mater. Sci..

[24] Aykol M (2019). Network analysis of synthesizable materials discovery. Nat. Commun..

[25] Bartel CJ (2020). A critical examination of compound stability predictions from machine-learned formation energies. npj Comput. Mater..

[26] Pickard CJ, Needs R (2011). Ab initio random structure searching. J. Phys. Condens. Matter.

[27] Wang H-C, Botti S, Marques MA (2021). Predicting stable crystalline compounds using chemical similarity. npj Comput. Mater..

[28] Hestness, J. et al. Deep learning scaling is predictable, empirically. Preprint at https://arxiv.org/abs/1712.00409 (2017).

[29] Furness JW, Kaplan AD, Ning J, Perdew JP, Sun J (2020). Accurate and numerically efficient r^2^SCAN meta-generalized gradient approximation. J. Phys. Chem. Lett..

[30] Batzner S (2022). E(3)-equivariant graph neural networks for data-efficient and accurate interatomic potentials. Nat. Commun..

[31] Thomas, N. et al. Tensor field networks: rotation- and translation-equivariant neural networks for 3D point clouds. Preprint at https://arxiv.org/abs/1802.08219 (2018).

[32] Togo, A. & Tanaka, I. Spglib: a software library for crystal symmetry search. Preprint at https://arxiv.org/abs/1808.01590 (2018).

[33] Behler J (2015). Constructing high-dimensional neural network potentials: a tutorial review. Int. J. Quantum Chem..

[34] Kresse G, Furthmüller J (1996). Efficient iterative schemes for ab initio total-energy calculations using a plane-wave basis set. Phys. Rev. B.

[35] Battaglia, P. W. et al. Relational inductive biases, deep learning, and graph networks. Preprint at https://arxiv.org/abs/1806.01261 (2018).

[36] Gilmer J, Schoenholz SS, Riley PF, Vinyals O, Dahl GE (2017). Neural message passing for quantum chemistry. Proc. Mach. Learn. Res..

[37] Chen C, Ye W, Zuo Y, Zheng C, Ong SP (2019). Graph networks as a universal machine learning framework for molecules and crystals. Chem. Mater..

[38] Kaplan, J. et al. Scaling laws for neural language models. Preprint at https://arxiv.org/abs/2001.08361 (2020).

[39] Hicks D (2021). AFLOW-XtalFinder: a reliable choice to identify crystalline prototypes. npj Comput. Mater..

[40] Blöchl PE (1994). Projector augmented-wave method. Phys. Rev. B.

[41] Perdew JP, Ernzerhof M, Burke K (1996). Rationale for mixing exact exchange with density functional approximations. J. Chem. Phys..

[42] Kitchaev DA (2016). Energetics of MnO_2_ polymorphs in density functional theory. Phys. Rev. B.

[43] Kingsbury R (2022). Performance comparison of r^2^SCAN and SCAN metaGGA density functionals for solid materials via an automated, high-throughput computational workflow. Phys. Rev. Mater..

[44] Bassman Oftelie L (2018). Active learning for accelerated design of layered materials. npj Comput. Mater..

[45] Cheon G (2017). Data mining for new two- and one-dimensional weakly bonded solids and lattice-commensurate heterostructures. Nano Lett..

[46] Sendek AD (2017). Holistic computational structure screening of more than 12000 candidates for solid lithium-ion conductor materials. Energy Environ. Sci..

[47] Behler J, Parrinello M (2007). Generalized neural-network representation of high-dimensional potential-energy surfaces. Phys. Rev. Lett..

[48] Bartók AP, Payne MC, Kondor R, Csányi G (2010). Gaussian approximation potentials: the accuracy of quantum mechanics, without the electrons. Phys. Rev. Lett..

[49] Lot R, Pellegrini F, Shaidu Y, Küçükbenli E (2020). PANNA: properties from artificial neural network architectures. Comput. Phys. Commun..

[50] Zhou Y, Qiu Y, Mishra V, Mar A (2021). Lost horses on the frontier: K_2_BiCl_5_ and K_3_Bi_2_Br_9_. J. Solid State Chem..

[51] Abudurusuli A (2021). Li_4_MgGe_2_S_7_: the first alkali and alkaline-earth diamond-like infrared nonlinear optical material with exceptional large band gap. Angew. Chem. Int. Ed..

[52] Ruan B-B, Yang Q-S, Zhou M-H, Chen G-F, Ren Z-A (2021). Superconductivity in a new *T*_2_-phase Mo_5_GeB_2_. J. Alloys Compd..

[53] Guo Z (2021). Local distortions and metal–semiconductor–metal transition in quasi-one-dimensional nanowire compounds AV_3_Q_3_O_*δ*_ (A = K, Rb, Cs and Q = Se, Te). Chem. Mater..

[54] Deng A (2021). Novel narrow-band blue light-emitting phosphor of Eu^2+^-activated silicate used for WLEDs. Dalton Trans..

[55] Zhak O, Köhler J, Karychort O, Babizhetskyy V (2022). New ternary phosphides *RE*_5_Pd_9_P_7_ (*RE*=Tm, Lu): synthesis, crystal and electronic structure. Z. Anorg. Allg. Chem..

[56] Zuo Y (2020). Performance and cost assessment of machine learning interatomic potentials. J. Phys. Chem. A.

[57] Davies DW (2019). SMACT: semiconducting materials by analogy and chemical theory. J. Open Source Softw..

[58] Goodall RE, Lee AA (2020). Predicting materials properties without crystal structure: deep representation learning from stoichiometry. Nat. Commun..

[59] Kresse G, Furthmüller J (1996). Efficiency of ab-initio total energy calculations for metals and semiconductors using a plane-wave basis set. Comput. Mater. Sci..

[60] Kresse G, Joubert D (1999). From ultrasoft pseudopotentials to the projector augmented-wave method. Phys. Rev. B.

[61] Mathew K (2017). atomate: a high-level interface to generate, execute, and analyze computational materials science workflows. Comput. Mater. Sci..

[62] Chen C, Ong SP (2022). A universal graph deep learning interatomic potential for the periodic table. Nat. Comput. Sci..

[63] Schoenholz S, Cubuk ED (2020). JAX MD: a framework for differentiable physics. Adv. Neural Inf. Process. Syst..

[64] Abadi, M. et al. TensorFlow: large-scale machine learning on heterogeneous systems. https://www.tensorflow.org/ (2015).

[65] Momma K, Izumi F (2011). VESTA 3 for three-dimensional visualization of crystal, volumetric and morphology data. J. Applied Crystallogr..

[66] Geiger, M. & Smidt, T. e3nn: Euclidean neural networks. Preprint at https://arxiv.org/abs/2207.09453 (2022).

[67] Anderson JA, Glaser J, Glotzer SC (2020). HOOMD-blue: a Python package for high-performance molecular dynamics and hard particle Monte Carlo simulations. Comput. Mater. Sci..

[68] Hendrycks, D. & Gimpel, K. Gaussian Error Linear Units (GELUs). Preprint at https://arxiv.org/abs/1606.08415 (2016).

[69] Jun K (2022). Lithium superionic conductors with corner-sharing frameworks. Nat. Mater..

[70] Ong SP (2013). Phase stability, electrochemical stability and ionic conductivity of the Li_10±1_MP_2_X_1_2 (M = Ge, Si, Sn, Al or P, and X = O, S or Se) family of superionic conductors. Energy Environ. Sci..

[71] Mo Y, Ong SP, Ceder G (2012). First principles study of the Li_1_0GeP_2_S_1_2 lithium super ionic conductor material. Chem. Mater..

