# Supplementary material for: Scaling deep learning for materials discovery
Source: Nature. 2023 Nov 29;624(7990):80–5. doi: 10.1038/s41586-023-06735-9 (PMC10700131; doi:10.1038/s41586-023-06735-9)
Supplement: Supplementary file 1 — The supplementary information contains six sections, providing further context to the computational experiments performed. [file 41586_2023_6735_MOESM1_ESM.pdf]

nature portfolio

https://doi.org/10.1038/s41586-023-06735-9

Supplementary information

Scaling deep learning for materials discovery

In the format provided by the authors and unedited

Supplementary Information for: Scaling Materials Discovery with Deep Learning

Amil Merchant*[1,2], Simon Batzner*[1], Samuel S. Schoenholz*[1], Muratahan Aykol[1], Gowoon Cheon[3], and Ekin Dogus Cubuk*[1]

[1]Google DeepMind
[2]Institute for Computational and Mathematical Engineering, Stanford University
[3]Google Research

*Equal contribution.

1 Supplementary Note 1: Additional information on Materials Discovery Results

1.1 Details of Experimental Matches

In Fig. 1 we provide additional summary statistics of matched experimental crystals as aggregated by ICSD, as described in the main body of the paper. In Fig. 1a, we provide the journal year, and in Fig. 1b and c, we showcase that experimental matches occur across a variety of unique element sizes and number of atoms. The tails of both figures in finding quaternary / quintary structures as well as structures whose reduced formula is greater than 20 supports the usage machine-learning guidance in these more complex search spaces. Finally, in Fig. 1d, we highlight the final computed decomposition energies of the experimental structures. The findings suggest even metastable structures can be of interest for experimental applications, further supporting the complete GNoME dataset beyond materials that are only on the convex hull.

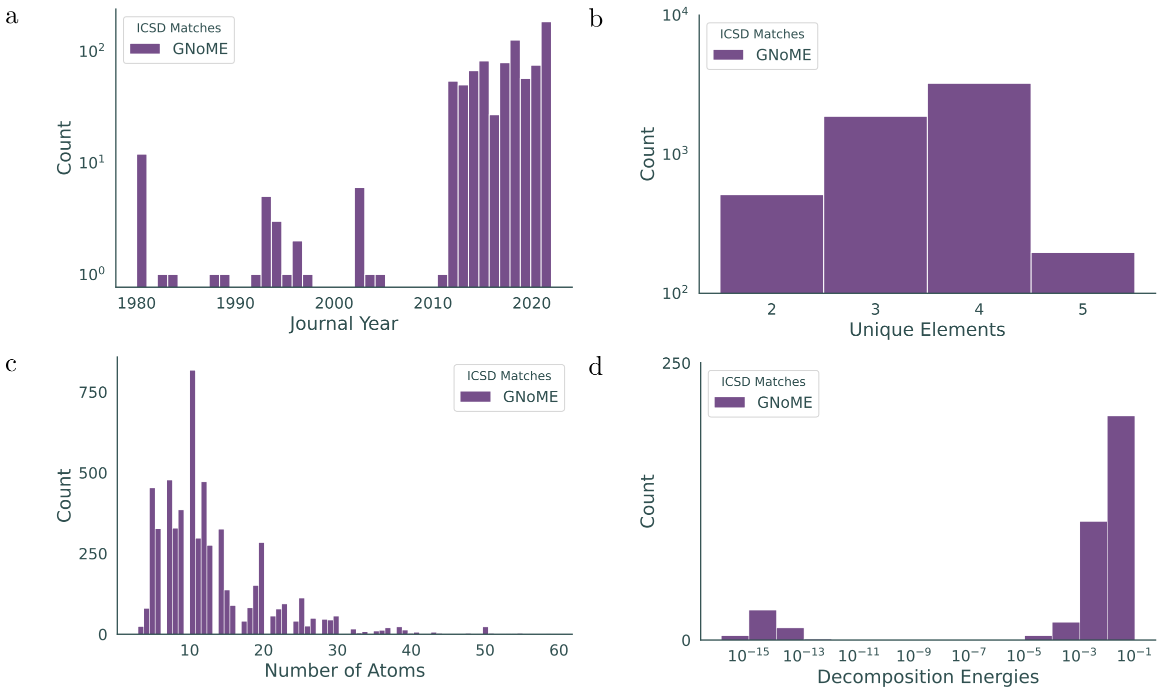

Supplemental Figure 1: GNoME discoveries match experimental discoveries aggregated by the Inorganic Crystal Structure Database (ICSD). We present a number of summary statistics in the figures above, including the journal year, number of unique elements, and number of atoms. In the final plot, we plot the decomposition energy for all of the experimental matches.

1.2 Patterns in Formation Energies

Prior work [1, 2] has postulated that stable materials with increasing number of elements should display increasingly negative formation energies. With the intuition that increasing the number of elements leads to a combinatorial increase in the number of competing phases, Fig. 2 displays distribution of formation energies for the stable materials in reproductions of Materials Project and OQMD in comparison to the GNoME dataset, indexed by the unique number of elements. While the same trend is observed in the GNoME dataset, the effect is significantly less pronounced. In addition, due to natural occurrence and research interests, current databases contain a relatively high population of highly ionic and covalent compounds with stronger bonds (e.g. oxides, fluorides etc.) yielding deeper formation energies. Our exploration in GNoME was not biased towards any particular chemistry and hence expanded into a wide range of chemical spaces where bonds are naturally not as strong (e.g. pnictides, chalcogenides, borides, alloys etc.), yielding formation energies that are not necessarily as

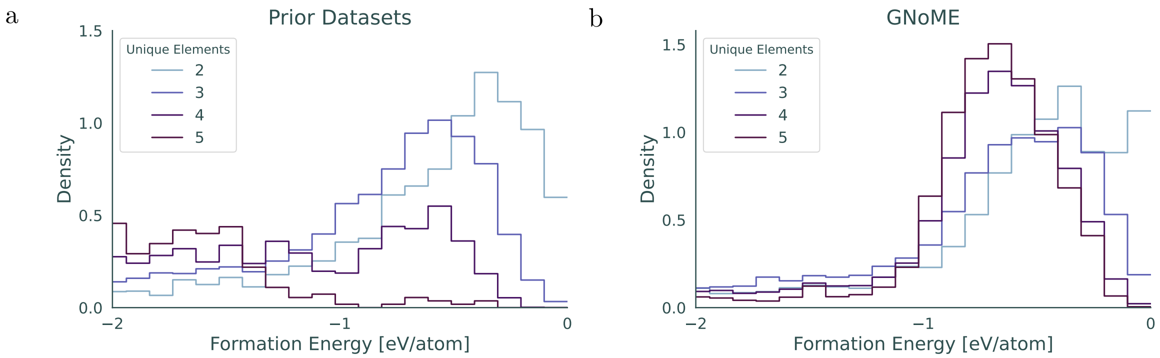

Supplemental Figure 2: Formation energies of all stable materials reproduced from Materials Project and OQMD compared to the discoveries by GNoME. We note the drastic difference in distribution, suggesting that GNoME models are able to discover more complex patterns of stability.

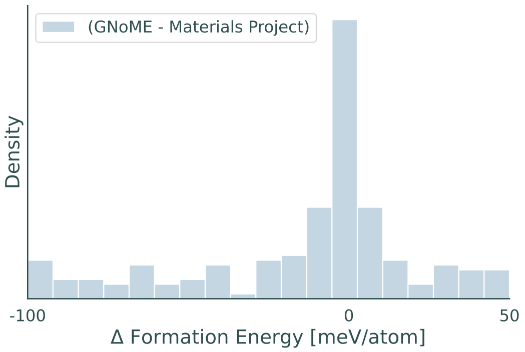

Supplemental Figure 3: Difference in formation energies when using the GNoME pipeline to find improved structures for compositions already available in prior catalogs.

deep.

1.3 Repeat Structure Comparisons

In the rest of this paper, we report results for compositions that are independent from downloaded databases such as Materials Project and OQMD [3, 4]. This decision ensures that the new structures discovered in this work are not slight modifications of already known materials, but are clearly novel. However, it is also known that the Materials Project [3] contains DFT calculations that are not intended to be global minima within a composition, for example, with the addition of many nano-wire structures between 2018 and 2019. There are also many compositions with only 1 viable structure, potentially indicating limited search heuristics used to find global minima.

Therefore, repeating structure searches on compositions already in the Materials Project and OQMD using GNoME models allows us to assess model capabilities for discovering better structures for compositions lacking the global minima in these databases. Fig. 3 presents the relative formation energy difference for these repeated computation. While many of the compositions do appear to be at global minima structures (indicating the success of years of research into specific families of interest), there is a notable number of compositions that improve. For the main body of the paper, these "repeated" composition comparisons are included in the phase diagram calculations for the updated convex hull but are not counted in the presented 381,000 novel stable crystal structures due to limitations in available crystal structure matchers.

1.4 Data Limitations

In this paper, we are consistent with prior efforts in the structure prediction community to count stable materials relative to the known convex hull. Future discoveries can always displace crystal structures

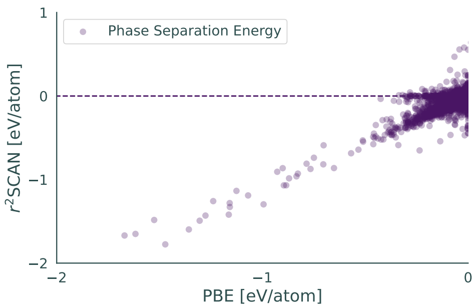

Supplemental Figure 4: Comparison of phase separation energies from the PBE and r^{2}SCAN functional for material structures directly from Materials Project or OQMD.

that are currently on the convex hull. For example, the GNoME discoveries displace approximately 5,000 of the "stable" structures from Materials Project and OQMD. The *true* convex hull will remain a continual goal in material science.

2 Supplementary Note 2: r^{2}SCAN Comparison

In the body of the paper, we validate the model predictions and materials discovered by the GNoME procedure by comparing energies between the commonly used PBE functionals and the more accurate but computationally expensive r^{2}SCAN procedure. The overall agreement is high, with 84% of structures marked as stable via PBE calculated as stable via r^{2}SCAN. Nevertheless, there is a somewhat noticeable discrepancy in phase separation energies near for elements close to the convex hull. Results from additional analysis are provided below.

First, the discrepancy in phase separation energies near 0 appears standard and not a unique feature of the GNoME dataset. In particular, Fig 4 plots an equivalent graph comparing the phase separation energies of crystals available in the Materials Project and OQMD suggesting that the misclassification via PBE is also likely for public dataset. We note that calculations at the level of r^{2}SCAN are also often intermediates between PBE and experimentation so the described errors in calculation are often fixed in preprocessing for downstream applications. As an exploratory measure, we have trained graph neural networks to explore transferring between PBE to r^{2}SCAN energies; early results suggest that this delta can be predicted to within 13meV/atom. Due to the computational expense of r^{2}SCAN compuations especially when not near local minima, this strategy has not yet been used for material discovery under the associated meta-GGA functional.

3 Supplementary Note 3: Patterns of Phase Separation

In Fig. 5, we provide greater context to the discussion surrounding phase separation energies of discoveries, to add to the quaternaries presented in the main body of the paper. There are improvements at all phase separation levels suggesting many structures found are meaningfully stable with respect to competing phases. Improvements to binaries are interesting as GNoME is able to find novel structures despite the simplicity and long-history of discovery in this space. Discoveries only increase in relative magnitude for ternaries and beyond.

3.1 Compositions Close to the Convex Hull

So far, we have placed predominant focus on compositions that are stable, as measured by being within floating point error ($1e-7$) of the convex hull. This stringent definition, however, neglects problems with the numeric instability of density functional theory results as well as the error in estimating experimental energies via computational methods. Additionally, inorganic crystals may be metastable and viable for industrial applications though not being the ground state of a particular composition, for example the diamond form of carbon in comparison to graphite. Therefore, experimentalists hoping to produce inorganic crystals will include candidates that are close to convex hull. In Fig. 6, we

a b c d

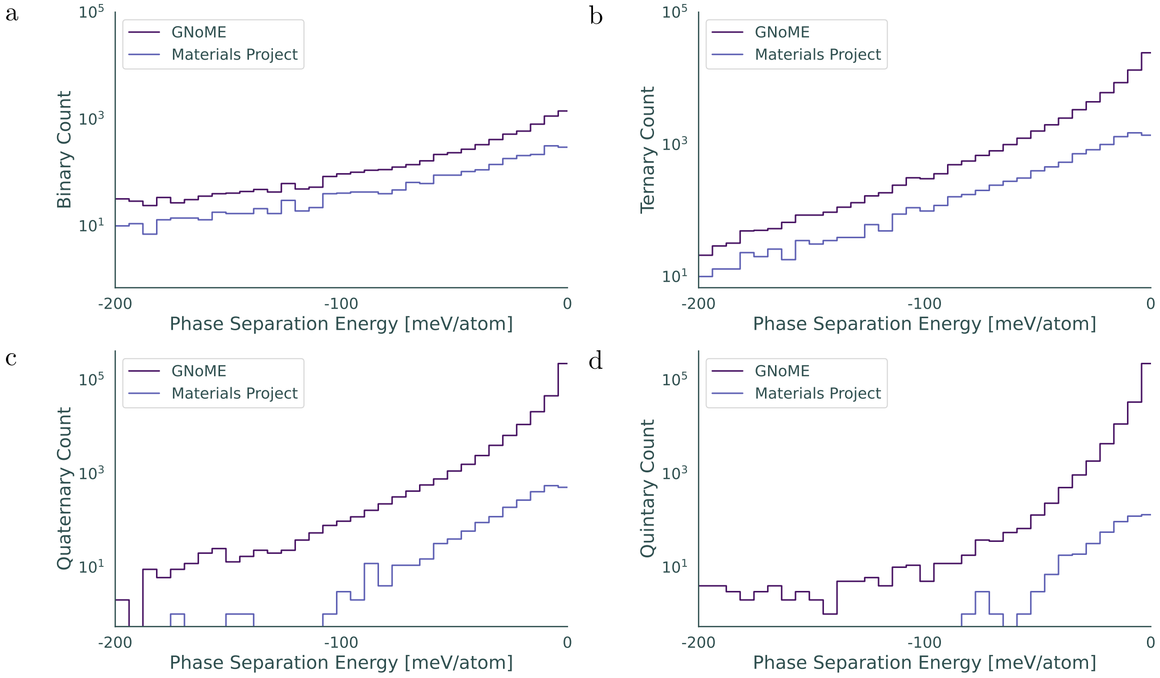

Supplemental Figure 5: Phase separation energies filtered by unique number of atoms on the complete GNoME dataset.

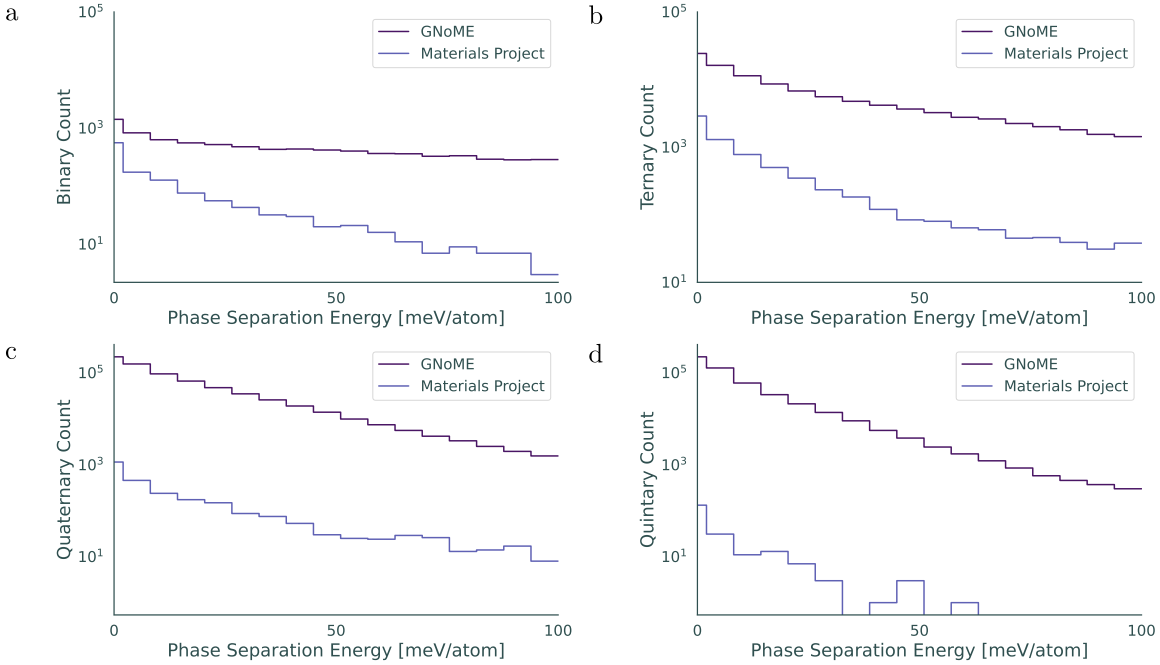

Supplemental Figure 6: Phase separation energies filtered by unique number of atoms on the complete GNoME dataset.

a

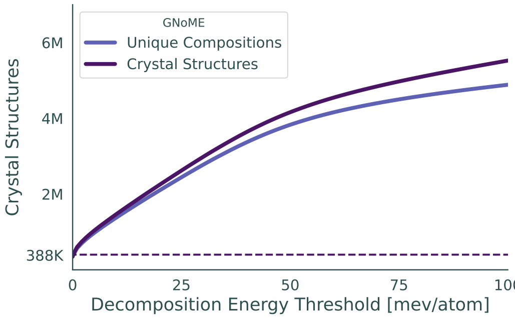

b

Threshold	0	25	50	100
Unique Compositions	388K	2.4M	3.8M	4.8M
Crystal Structures	388K	2.4M	4.1M	5.5M

Supplemental Figure 7: A summary of the GNoME dataset, filtered by distance to the convex hull.

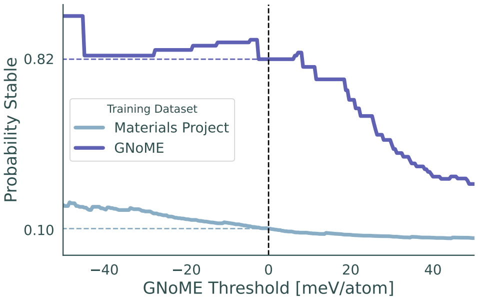

Supplemental Figure 8: Precision of machine learning models trained on GNoME vs. the Materials Project data, as a function of the prediction threshold.

showcase how the active learning process also introduces a significant number of the discoveries made by GNoME lie close to the convex hull, providing an even greater set of candidates for potential downstream applications. A promising avenue for future work would be to look into patterns of metastablility and contrast with the stable materials discovered.

Fig. 7 further summarizes the counts of unique compositions or independent crystal structures that are close to the convex hull by filtering by decomposition energy. In particular, we find that with common thresholds of 25 or 50 meV/atom from the convex hull, the GNoME dataset provides a substantially large number of crystal structures. Even at the more conservative 25 meV/atom metric, there are over 2.4M unique compositions and associated structures in the GNoME dataset.

3.2 Filtration Improvement from Active Learning

In the main body of the paper, we reference the increasing hitrate as models improve via active learning. In Fig. 8, we break down the hitrate of GNoME models with respect to the threshold chosen. On subsets of materials generated from the initial round of active learning and the final round, we evaluate model performance and show that despite equivalent filters (e.g. with test-time augmentation and uncertainty quantification), GNoME models show improved performance across the board. Furthermore, the results suggest that a fraction of GNoME discoveries come from substitutions that would not have been immediately predicted as stable (before relaxation).

3.3 Remaining Materials

An open research question after the GNoME discoveries is how many materials remain to be discovered? In the paper, we discussed the results from 6 rounds of active learning, resulting models of high hitrate overall. Preliminary results suggest that a great deal more materials remain to be found, with active learning on the start of a 7th round and extrapolations of the hitrate suggesting at least an additional

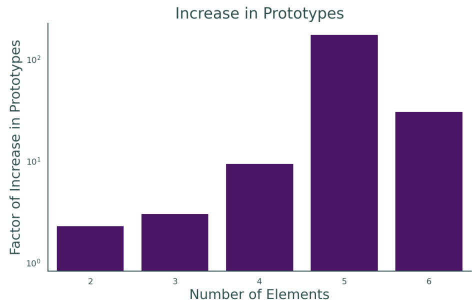

Supplemental Figure 9: Increase in the number of prototypes as a ratio to those available by the Materials Project.

1.6M (80% * 2 million candidates) materials that are likely to be stable with respect to the convex hull of Materials Project. It is likely some of these would drive up the number of stable crystals on the updated hull. These results suggest that GNoME can continue to aid in discovery either via high-throughput search or within specific families of interest.

3.4 Additional Prototype Analysis

Prototype analysis in the main body of the paper is presented in a log-scale making the relative contributions to individual number of elements difficult to ascertain. In Fig. 9, we present the relative increase in the number of prototypes across number of elements. This figure emphasizes the capabilities of GNoME models for discovering materials with an increasing number of unique elements.

4 Supplementary Note 4: Analysis of 'Newly' Discovered Materials

4.1 Composition Matching

In our work, Materials Project, OQMD and WBM were used as reference databases against which we check if a new stable material we identify should be labeled as a stable discovery or not. First two of these databases include structures from comprehensive curated sources such as ICSD and Pauling File, and hence are considered to encompass nearly all experimentally known ordered inorganic structures. There could, however, still be known materials reported in the literature but overlooked in these databases. To understand the extent of this gap, we randomly sampled 200 structures from the stable GNoME discoveries, and ran independent and deep manual literature searches, factoring in human chemical knowledge (e.g. searching for other potential ways of writing the formula). Out of these 200, we found only one composition ($RbCaAsO_4$) that was in the literature but was not curated into the reference databases. Albeit not a large sample, these results imply that the number of false positive stable discoveries due to missing entries in reference databases is expected to be small.

4.2 Targeted Search

While we focused on efficiently finding new stable materials throughout this effort, convex-hull by its nature is never complete and a deeper search can always find new compounds that displace the older ones from the convex hull. To gauge the impact of this on the number of new stable materials we found, we randomly picked chemical system of 100 structures from our final list of 381,000 stable entries, and ran a more comprehensive structure search for each system, including the subspaces (e.g. if the target system is a ternary, we also include its binaries). In this deeper search, we generate candidate structures by using our substitutional pipeline on all of the prototypes available in GNoME for each of the 100 chosen chemical systems, which yields $>$ 10 million candidate structures. To downselect candidates for DFT, we further relax the filtering criteria used in our original pipeline, allowing (i) structures with a graph-network predicted energy within 100 meV/atom of our final convex hull and (ii) for each composition, up to 10 polymorphs to be included in the new batch of DFT calculations.

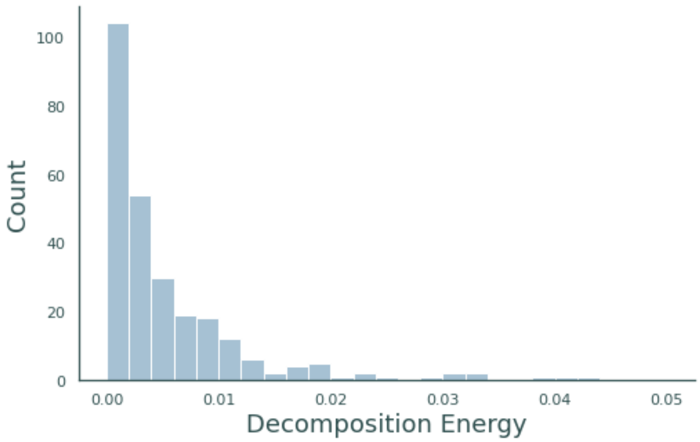

Supplemental Figure 10: Phase separation energies for materials removed from the convex hull by additional exploration within 6,500 selected chemical systems. Results showcase how stable materials can be displaced but often remain close to the updated convex hull. The results are likely still of interest to experimental researchers and for filtration in downstream applications.

This filtering yields approximately 100,000 new structures to calculate with DFT. After the DFT relaxations, we found that the total number of stable materials in these 100 chemical systems and their subspaces increased from 10,879 to 11,575. In addition, 270 materials were displaced from the convex-hull with this new search, for which we show the new decomposition energies remain close to the new hull (Fig. 10). These results indicate that while there is always room for finding new stable materials, our final convex hull provides a strong baseline for stabilities that do not significantly change with subsequent deeper searches.

5 Supplementary Note 5: Compositional model trained on AIRSS runs

As described in the methods section, we find that compositions for which more than 10 AIRSS runs have completed to be more reliable training and test labels for training compositional models. Fig. 11 shows that for compositions with fewer than 10 completed AIRSS runs, the decomposition energy can be anomalously large. Fig. 12 shows that while some compositional energy predictions from the GNN can seem too low, we find that these predictions almost always correspond to compositions for which fewer than 10 AIRSS runs have completed. When we restrict the comparison to compositions with at least 10 completed AIRSS runs, the agreement between the measured formation energies and predicted formation energies is much stronger.

6 Supplementary Note 6: GNoME Potential

6.1 Ionic conductivity prediction

Fig. 3 in the main text shows the performance of various pretrained MLIPs on the task of classifying whether an unseen material is a superionic conductor. These simulations are performed in a zero-shot manner, meaning that the pretrained GNoME potentials have neither seen any data sampled from AIMD, nor have been trained on the composition being simulated. In some cases, as common in MLIPs [5], one may encounter unstable simulations from which no measure of conductivity can be derived. When evaluating a model's ability to predict whether an unseen material displays superionic conductivity or not, including such simulations as misclassifications or discarding them from the test set may change the classifier performance. We report both. In Figure 3a in the main text, we report unstable simulations as not counting towards to the classification accuracy. In the supplementary information, in Fig. 13, we show the same experiment, but counting them as misclassified, thereby slightly increasing the classification error.

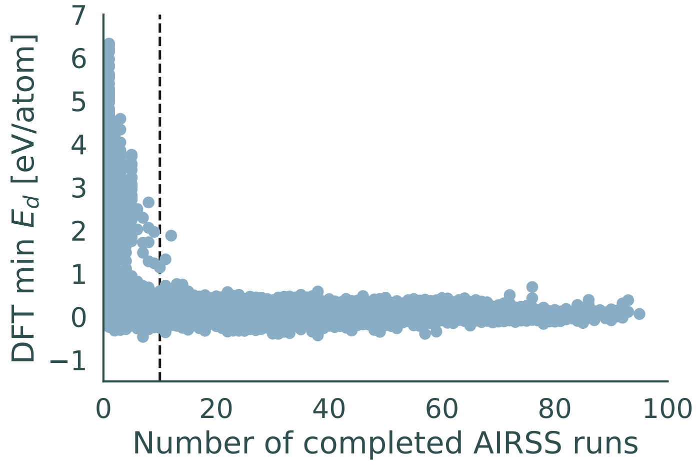

Supplemental Figure 11: Minimum decomposition energy for a composition as a function of AIRSS runs completed for that composition.

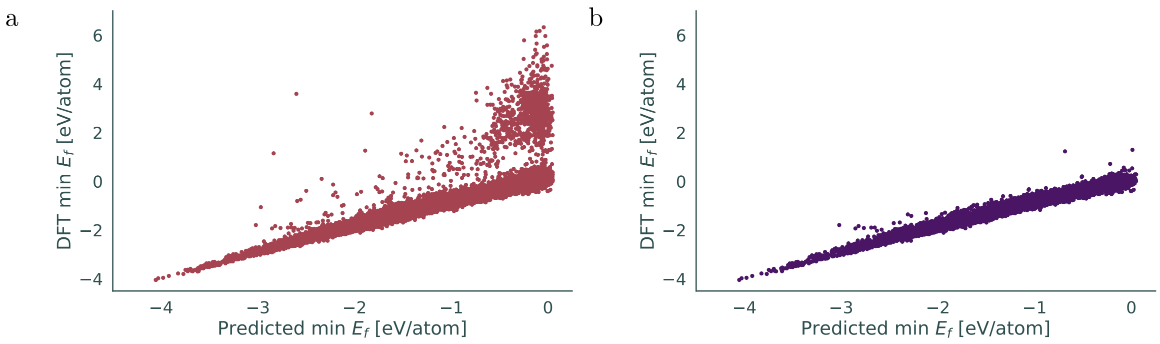

Supplemental Figure 12: (a) A scatter plot of formation energy labels from DFT and formation energy predictions from the compositional model. Each point represents a composition, and all compositions in our training set is included in the figure. (b) A scatter plot of formation energy labels from DFT and formation energy predictions from the compositional model. Each point represents a composition, but only compositions with at least 10 completed AIRSS runs are included.

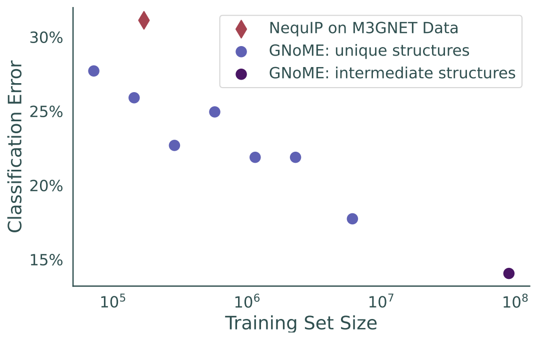

Supplemental Figure 13: The scaling of ionic conductivity classification error when the unstable GNN-MD simulations are considered to be classification errors. In contrast, in Fig. 3a the unstable GNN-MD simulations are considered as a no-prediction. We see that the general trends are similar for both assumptions.

6.2 Scaling behaviour of force errors on AIMD data

Deep learning has been empirically observed across various domains to exhibit predictable scaling behaviour, where the test error follows a power-law of the form $\epsilon = aN^b$, where ϵ and N are the predictive error and the number of training samples, respectively, and a and b are constants. Scaling laws have proven highly useful as they allow to predict what training set size would be required to obtain a given predictive error [6, 7]. We find that the mean absolute error in force components of downstream materials sampled from AIMD follows a power-law with respect to the pretraining size and consistently improves over *multiple decades*. Figures 14 - 21 show the scaling behaviour of the mean absolute error in force component as a function of training set size. Blue points represent scaling of the GNoME potential on unique structures from AIMD trajectories, red denotes a NequIP potential trained on the M3GNET data, and purple shows a GNoME potential trained on the full relaxation trajectory, including repeated structures along the minimization trajectory up to approx. 89 million structures. All four materials are compositions in the test set not included in training. We observe a clear power-law as we increase the pretraining data set size, for both T=400K and T=1,000K test evaluation.

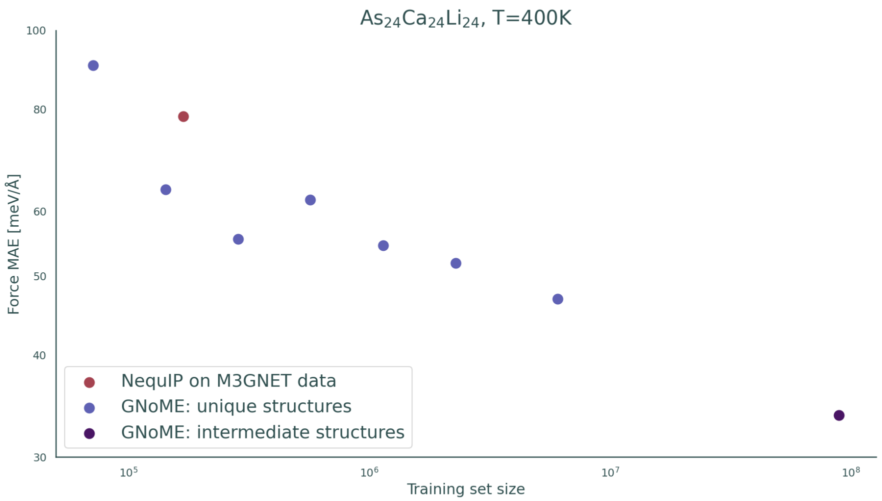

Supplemental Figure 14: **Force MAE, $As_{24}Ca_{24}Li_{24}$ at T=400K.**

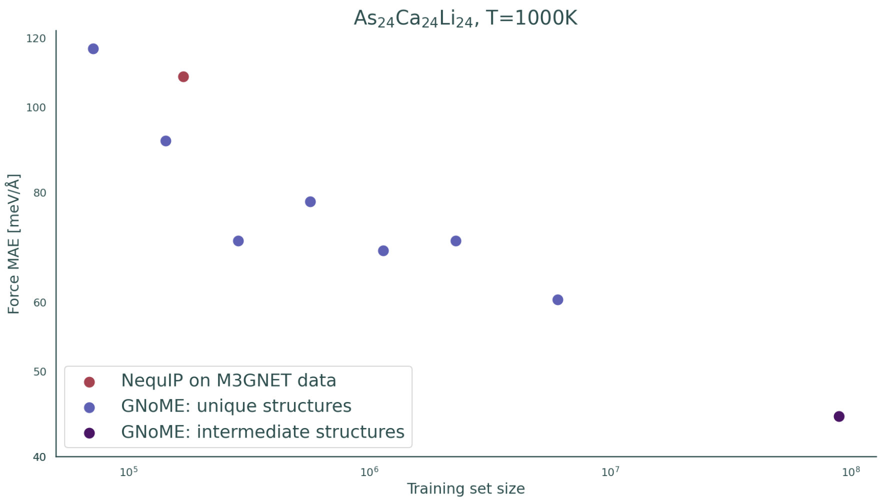

Supplemental Figure 15: **Force MAE, $As_{24}Ca_{24}Li_{24}$ at T=1,000K.**

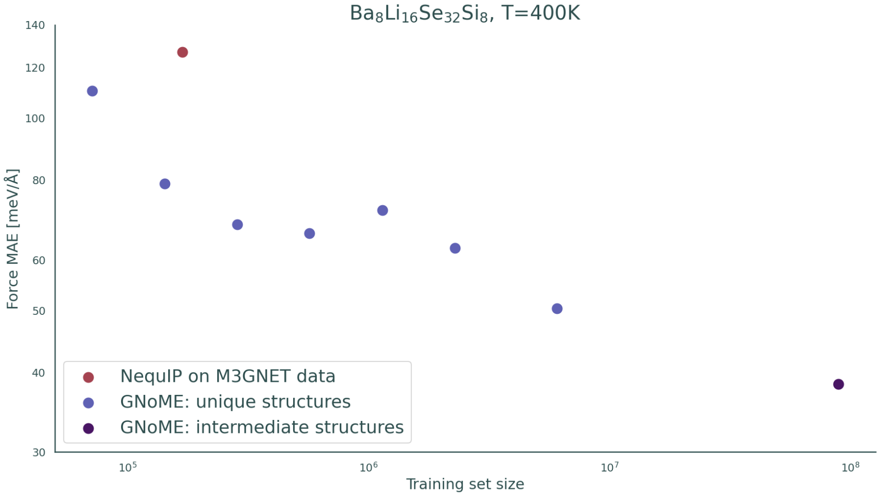

Supplemental Figure 16: **Force MAE, $Ba_8Li_{16}Se_{32}Si_8$ at T=400K.**

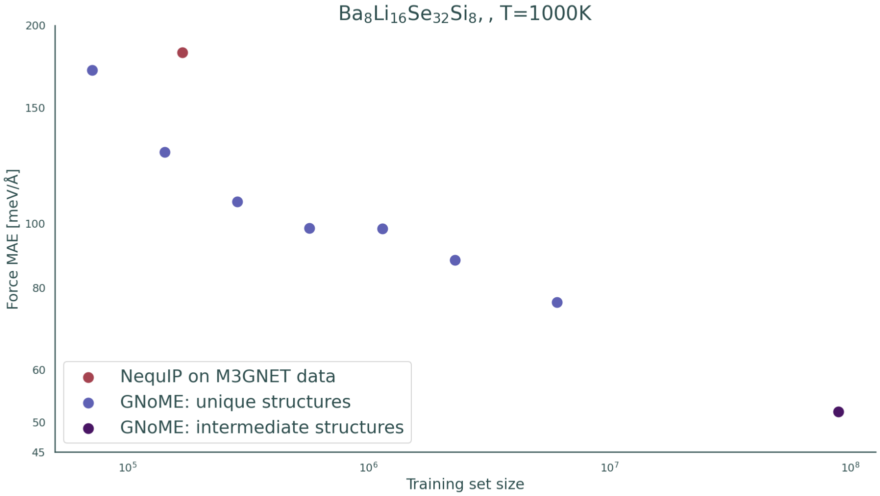

Supplemental Figure 17: **Force MAE, $Ba_8Li_{16}Se_{32}Si_8$ at T=1,000K.**

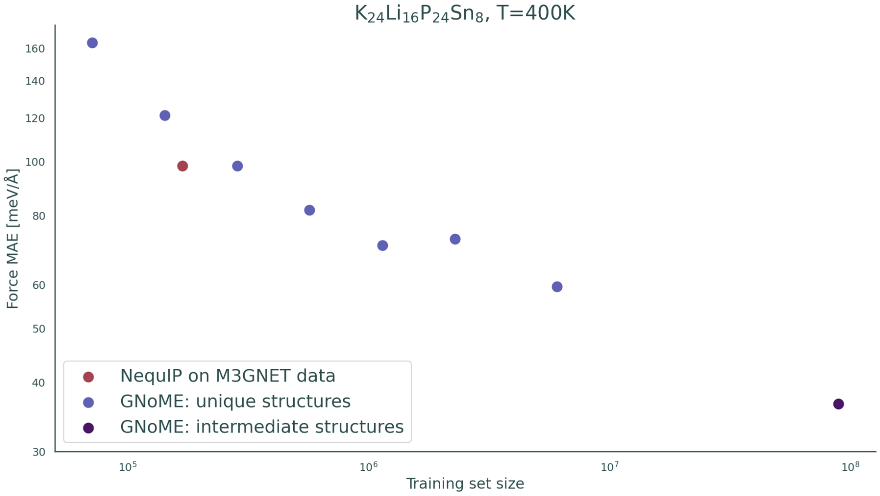

Supplemental Figure 18: **Force MAE, $K_{24}Li_{16}P_{24}Sn_8$ at T=400K.**

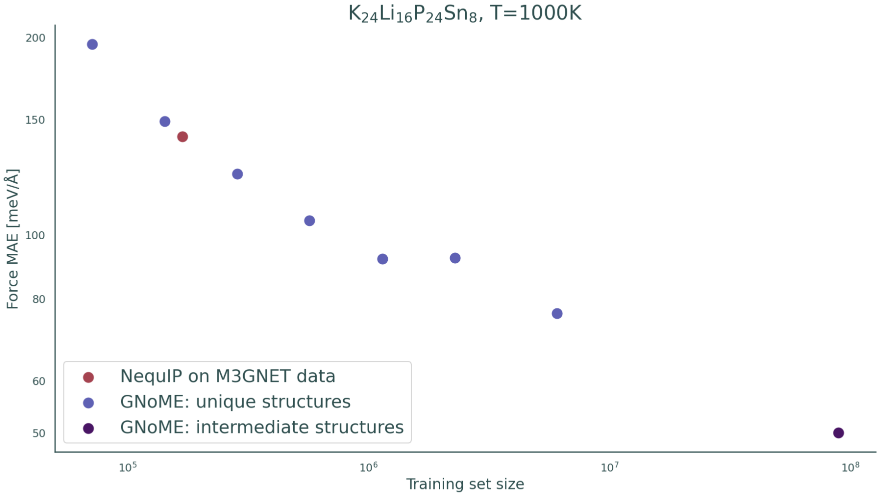

Supplemental Figure 19: **Force MAE, $K_{24}Li_{16}P_{24}Sn_8$ at T=1,000K.**

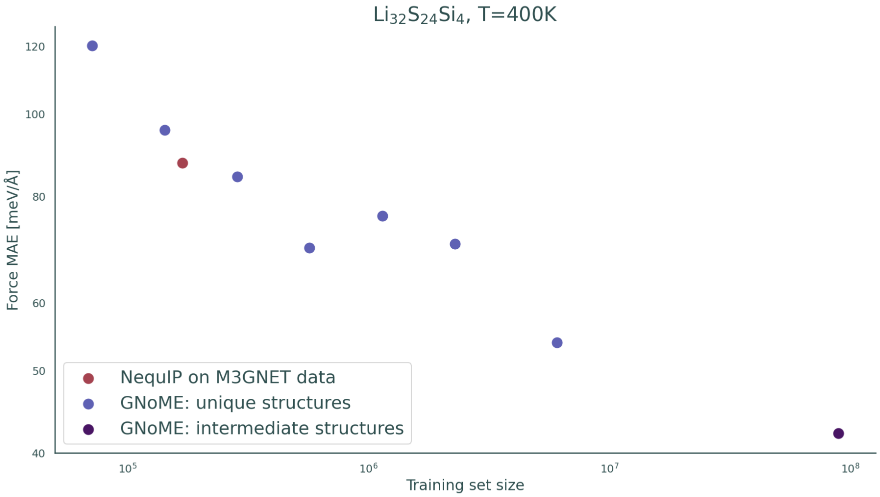

Supplemental Figure 20: **Force MAE, $Li_{32}S_{24}Si_4$ at T=400K.**

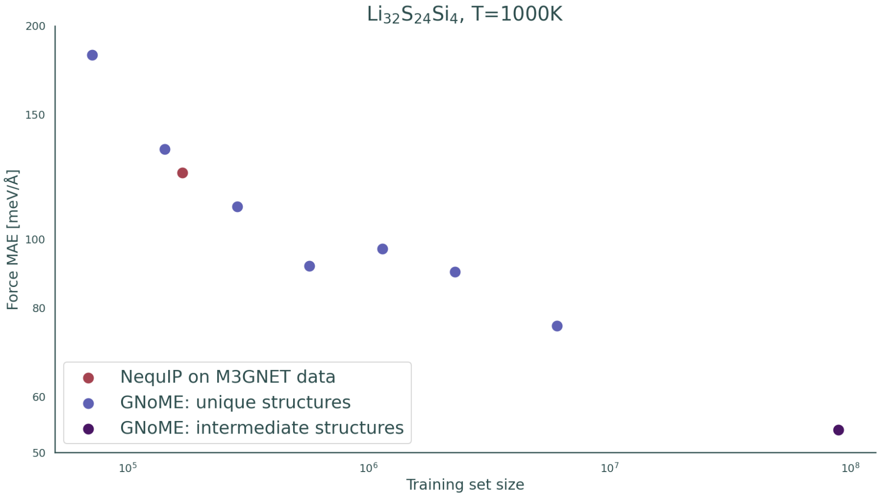

Supplemental Figure 21: **Force MAE, $Li_{32}S_{24}Si_4$ at T=1,000K.**

6.3 Robustness

A common shortcoming of MLIPs is their inability to generalize to data distributions beyond what they are trained on. This can result in unstable simulations or unphysical configurations, if configurations that lie outside of the training distribution are encountered during the simulation. One example of such domain shift is simulations with higher temperatures than the training set. We find that pretraining on the large and diverse GNoME dataset of structural relaxations greatly improves the robustness under such distribution shifts. In an effort to assess the model's robustness, we train two types of NequIP potentials on data of increasing sizes, sampled from AIMD simulations at T=400K and evaluate them on structures sampled at T=1,000K. As discussed in the Methods section, we train a) a NequIP potential starting from randomly initialized weights, and b) a model that was pretrained on the GNoME data set and then fine-tuned on the T=400K data. We also compare to the performance of the zero-shot model, that was never trained on the 400K data or the material composition to begin with. In figures 22 to 33, we demonstrate the performance of the potentials under a distribution shift by testing them on four different materials that are not included in training. For each composition, we first show a scatter plot of the performance at T=400K vs T=1,000K, where a perfect $y = x$ fit would indicate a perfectly robust potential. We find that pretraining consistently and substantially improves robustness. Second, we show the performance of the potentials trained from scratch and pretrained potentials as a function of fine-tuning data set size. We perform this experiment for evaluations at both 400K and 1,000K (note that in both cases, both the fine-tuning and training are performed at 400K). While in the 400K case, fine-tuning helps, the zero-shot model is quickly superseded in performance when training on data from the target distribution. The most significant improvement is observed on data sampled at T=1,000K where pretraining gives strong improvements, even at large data set size of $> 1,000$ structures. In addition, here the zero-shot model is also often highly competitive, often outperform from-scratch models trained on hundreds of samples.

Interestingly, we find that for only three out of the four materials in the test set, fine-tuning improves over zero-shot performance on robustness. Similarly, with large enough data sets, training from scratch improves upon zero-shot robustness. But for one material, neither is true. Analysis of the AIMD data reveals that while the three materials that improve are all poor conductors at T=1,000K, the remaining material exhibits Li-ion conduction at T=1,000K, but not at 400K. This suggests that in this case, overfitting on the non-conducting data hurts model performance when evaluated on the conducting case. The pretrained potential appears to perform better in a zero-shot evaluation.

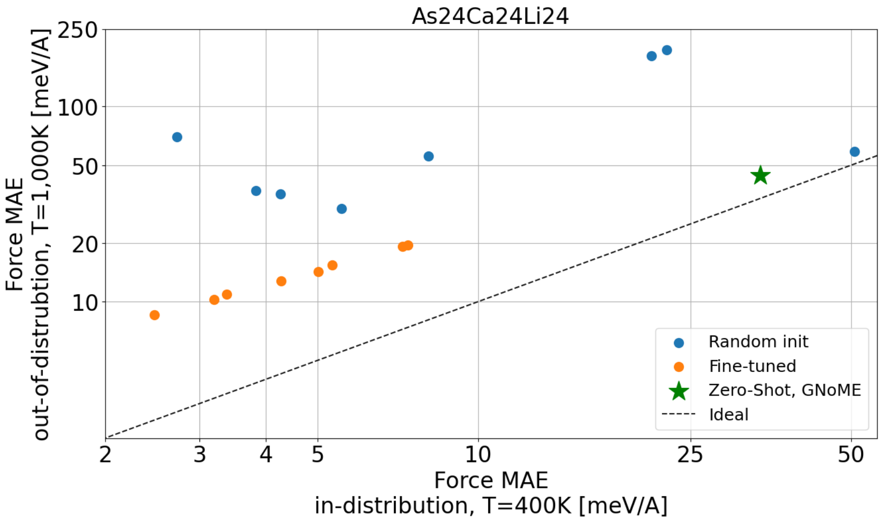

Supplemental Figure 22: **Robustness, $As_{24}Ca_{24}Li_{24}$**. In-domain vs out-of-domain error of different fine-tuned and randomly initialized models trained at T=400K and evaluated at T=400K as well as T=1,000K on.

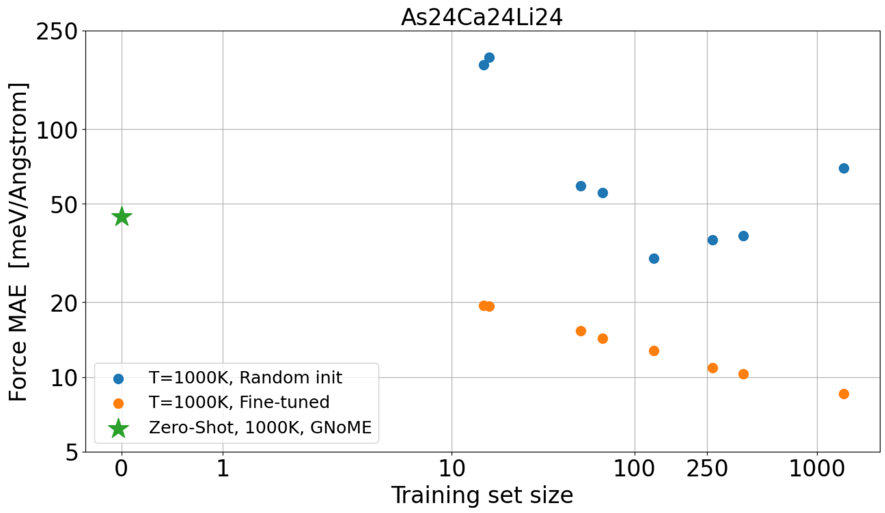

Supplemental Figure 23: **Robustness, $As_{24}Ca_{24}Li_{24}$, T=1,000K**. Force errors on data sampled at T=1,000K of fine-tuned and randomly initialized models trained at T=400K as a function of training set size

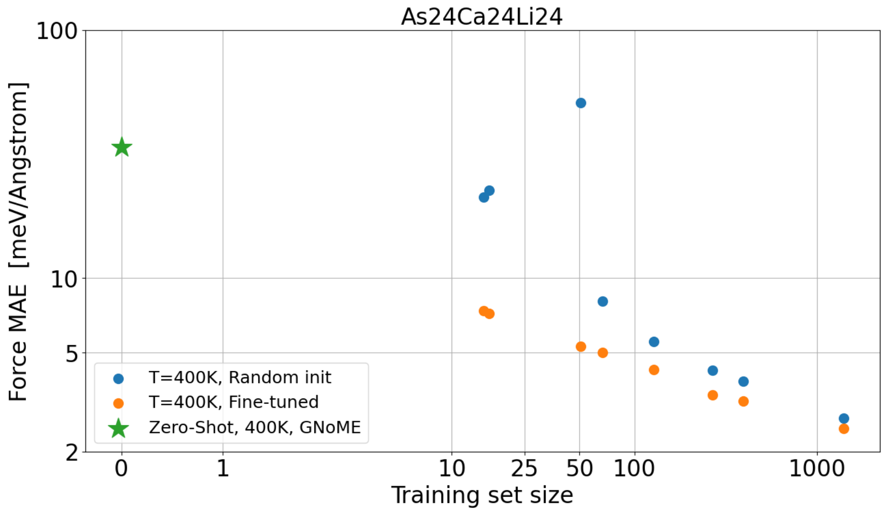

Supplemental Figure 24: **Robustness, $As_{24}Ca_{24}Li_{24}$, T=400K**. Force errors on data sampled at T=400K of fine-tuned and randomly initialized models trained at T=400K as a function of training set size

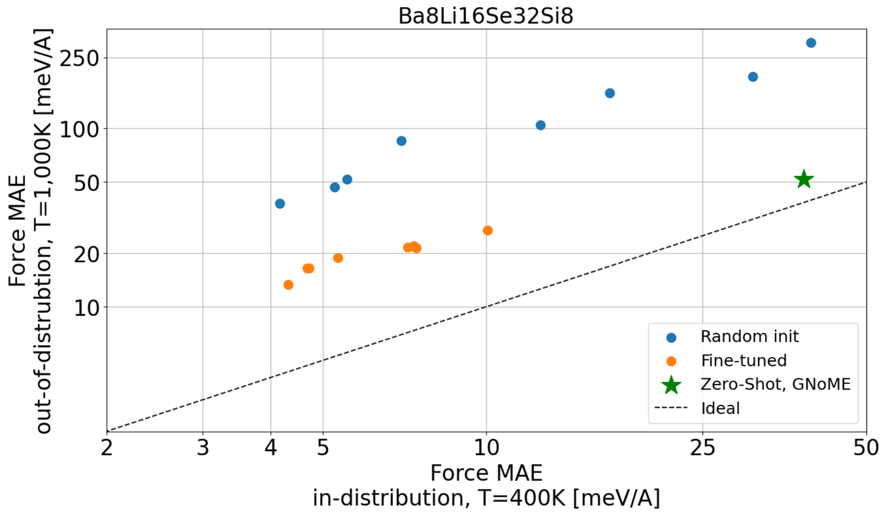

Supplemental Figure 25: **Robustness, $Ba_8Li_{16}Se_{32}Si_8$**. In-domain vs out-of-domain error of different fine-tuned and randomly initialized models trained at T=400K and evaluated at T=400K as well as T=1,000K on.

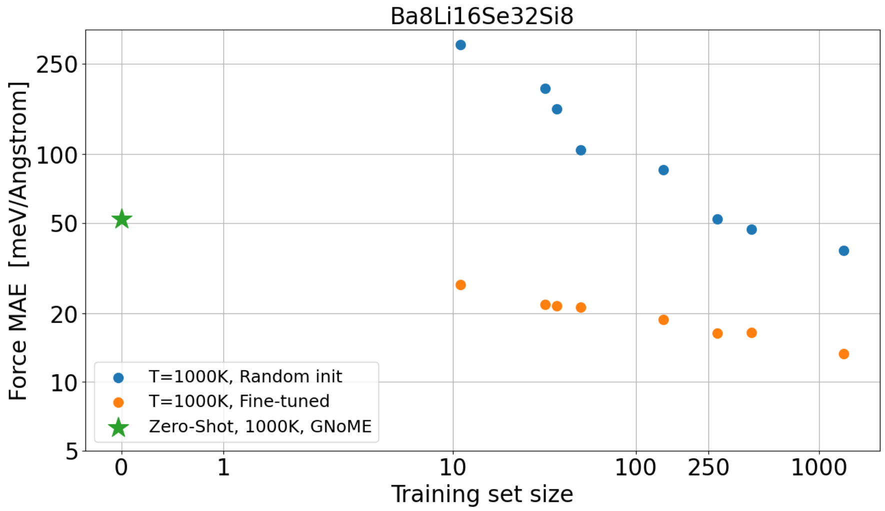

Supplemental Figure 26: **Robustness, $Ba_8Li_{16}Se_{32}Si_8$, T=1,000K**. Force errors on data sampled at T=1,000K of fine-tuned and randomly initialized models trained at T=400K as a function of training set size

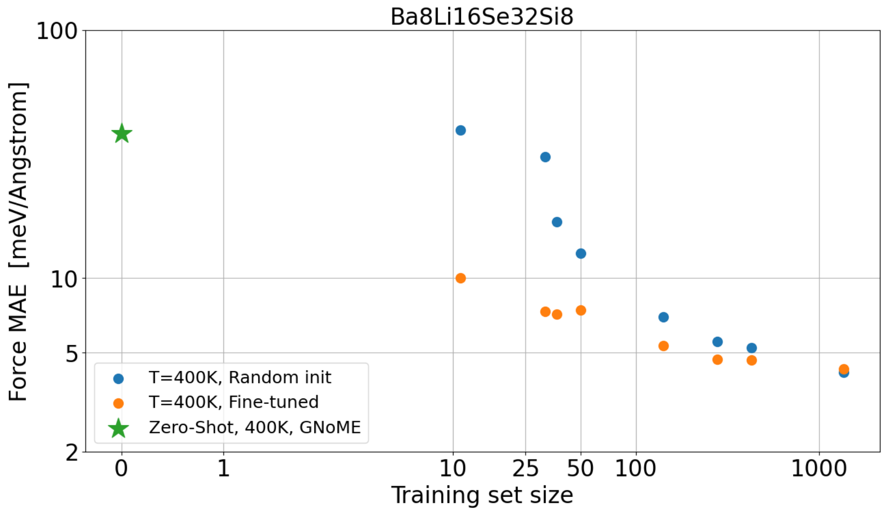

Supplemental Figure 27: **Robustness, $Ba_8Li_{16}Se_{32}Si_8$, T=400K**. Force errors on data sampled at T=400K of fine-tuned and randomly initialized models trained at T=400K as a function of training set size

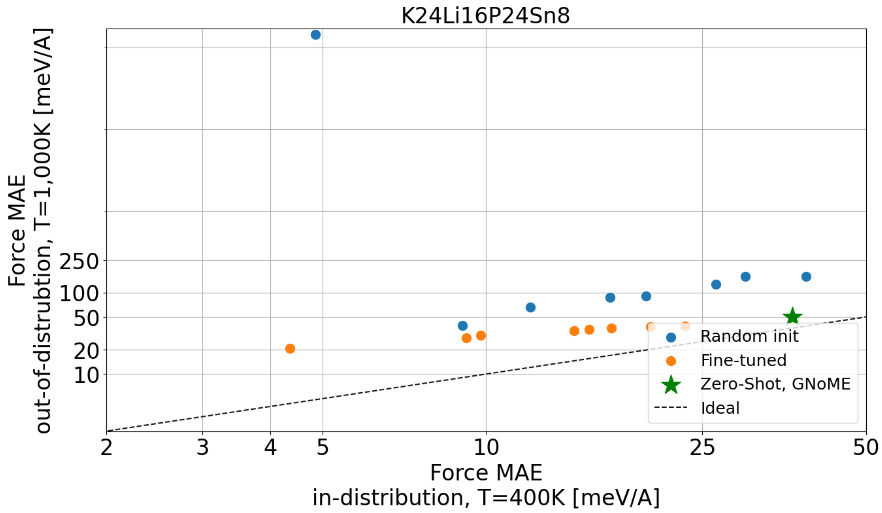

Supplemental Figure 28: **Robustness, $K_{24}Li_{16}P_{24}Sn_8$**. In-domain vs out-of-domain error of different fine-tuned and randomly initialized models trained at T=400K and evaluated at T=400K as well as T=1,000K on.

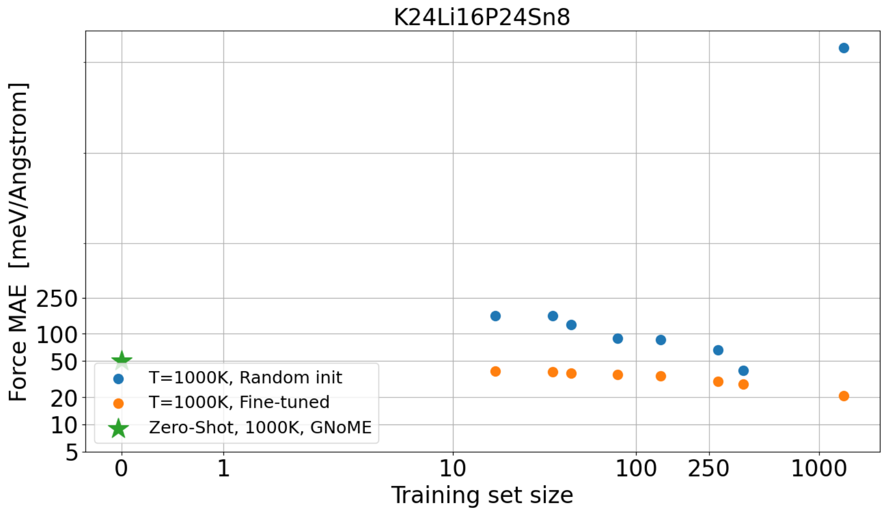

Supplemental Figure 29: **Robustness, $K_{24}Li_{16}P_{24}Sn_8$, T=1,000K**. Force errors on data sampled at T=1,000K of fine-tuned and randomly initialized models trained at T=400K as a function of training set size

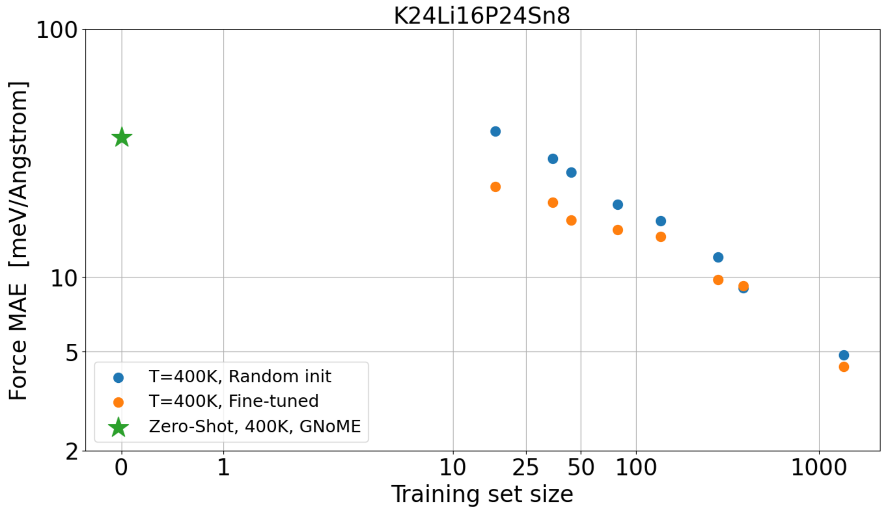

Supplemental Figure 30: **Robustness, $K_{24}Li_{16}P_{24}Sn_8$, T=400K**. Force errors on data sampled at T=400K of fine-tuned and randomly initialized models trained at T=400K as a function of training set size

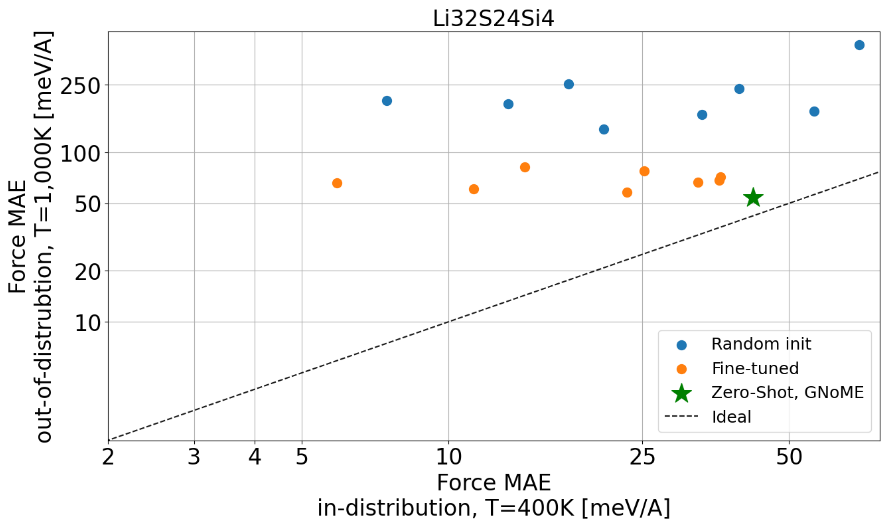

Supplemental Figure 31: **Robustness, $Li_{32}S_{24}Si_4$.** In-domain vs out-of-domain error of different fine-tuned and randomly initialized models trained at T=400K and evaluated at T=400K as well as T=1,000K on.

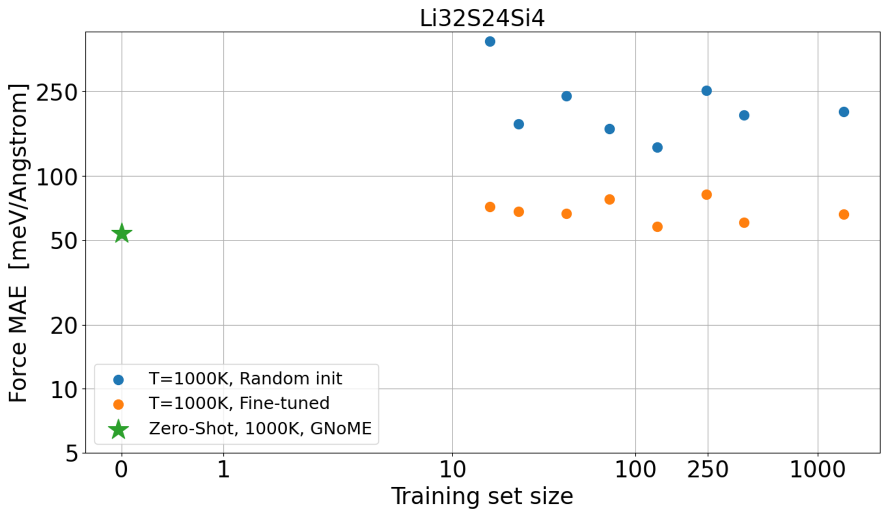

Supplemental Figure 32: **Robustness,$Li_{32}S_{24}Si_4$ T=1,000K**. Force errors on data sampled at T=1,000K of fine-tuned and randomly initialized models trained at T=400K as a function of training set size

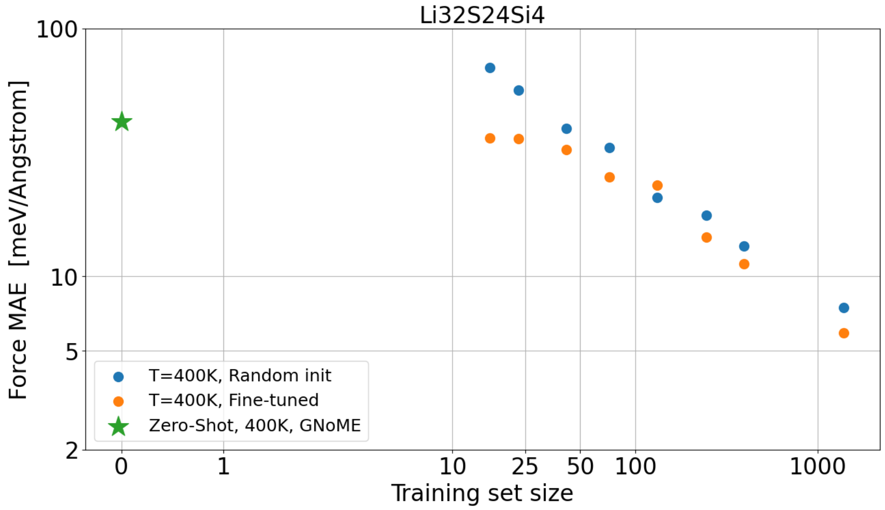

Supplemental Figure 33: **Robustness, $Li_{32}S_{24}Si_4$, T=400K**. Force errors on data sampled at T=400K of fine-tuned and randomly initialized models trained at T=400K as a function of training set size

6.4 Zero-Shot performance

6.4.1 Performance on structures sampled from AIMD

Figures 34 to 37 show the performance on structures sampled from AIMD runs of four unseen test set compositions that are randomly selected, sampled at 400K and 1000K. We observe strong performance on all four materials in the force errors, in particular in comparison to a NequIP model trained on the orders of magnitude smaller M3GNET data set (for a comparison with the M3GNET *potential*, as opposed to the dataset, see the section 6.4.2).

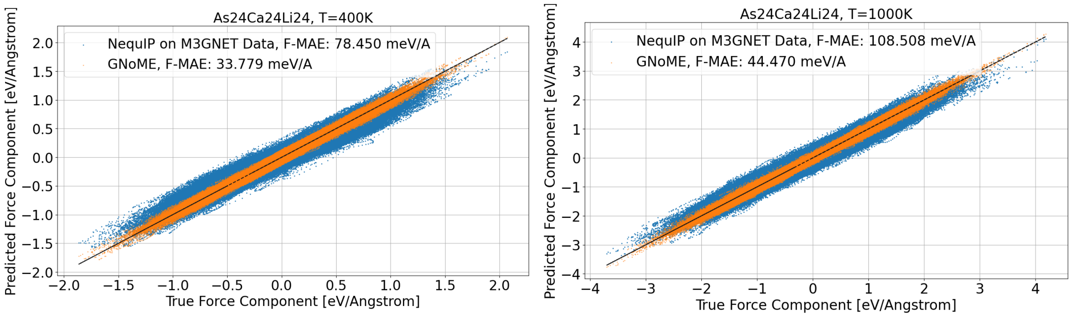

Supplemental Figure 34: **Performance of pretrained GNoME models on $As_{24}Ca_{24}Li_{24}$** Each plot also includes a comparison to a pretrained model trained on the M3GNET data. a) Pretrained, evaluated at T=400K b) Pretrained, evaluated at T=1,000K

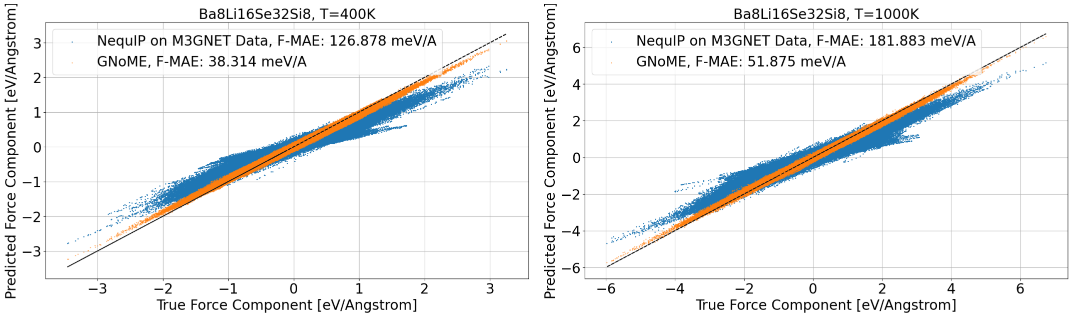

Supplemental Figure 35: **Performance of pretrained GNoME models on $Ba_8Li_{16}Se_{32}Si_8$** Each plot also includes a comparison to a pretrained model trained on the M3GNET data. a) Pretrained, evaluated at T=400K b) Pretrained, evaluated at T=1,000K

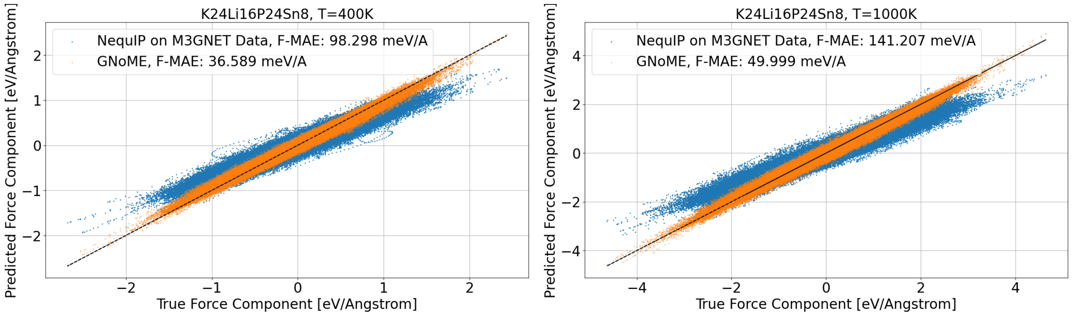

Supplemental Figure 36: **Performance of pretrained GNoME models on $K_{24}Li_{16}P_{24}Sn_8$** Each plot also includes a comparison to a pretrained model trained on the M3GNET data. a) Pretrained, evaluated at T=400K b) Pretrained, evaluated at T=1,000K

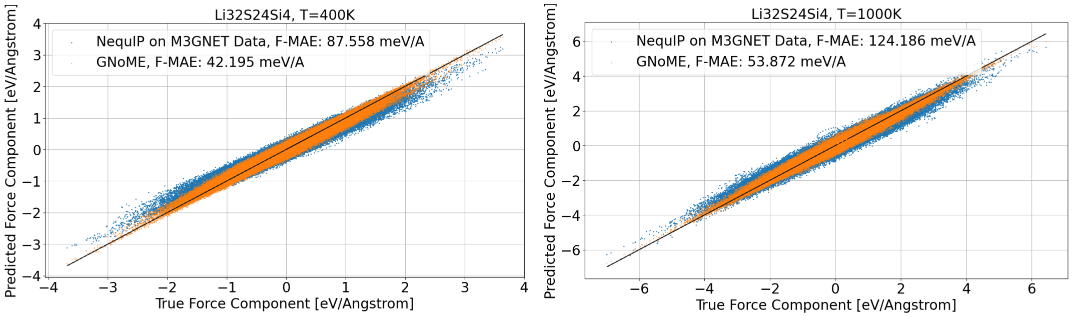

Supplemental Figure 37: **Performance of pretrained GNoME models on $Li_{32}S_{24}Si_4$** Each plot also includes a comparison to a pretrained model trained on the M3GNET data. a) Pretrained, evaluated at T=400K b) Pretrained, evaluated at T=1,000K

6.4.2 Performance on elemental materials

We further test the zero-shot performance of the GNoME potential on a series of elemental systems previously used in the performance assessment of machine learning interatomic potentials [8]. The data cover six elemental systems Li, Ni, Ge, Si, Cu, Mo, computed with DFT. The DFT computations were performed with VASP using the Perdew-Burke-Ernzerhof (PBE) generalized gradient approximation (GGA). The dataset from [8] covers a diverse set of structures including the ground state crystal structure, data sampled from NVT-AIMD at different temperatures (0.5x, 0.9x, 1.5x, and 2.0x the melting point), NVT-AIMD simulations of the bulk structure with a single vacancy at different temperatures (300K and 2.0x the melting point), strained structures, and slab structures. In [8], a series of MLIPs were trained on data from this distribution and tested on the same distribution. Here, we evaluate the pretrained GNoME potentials *directly* on the test set, without any training on the data. The data were obtained from the test set files from https://github.com/materialsvirtuallab/mlearn/tree/master/data.

Tables 1 - 6 show the zero-shot GNoME performance compared to different potentials trained on data from the same distribution, as well as to an evaluation of the pretrained, publicly available M3GNET and CHGNet potentials [9, 10]. We observe strong improvements from scaling up pretraining data sets. Moreover, we observe for the first time that a pretrained potential is competitive with machine learning interatomic potentials trained *explicitly* on the data. The pretrained GNoME potential is on par with a Behler-Parrinello neural network trained on hundreds of structures, outperforming it on four out of six materials. We note the special case of Nickel, where the composition is not present in our pretraining data, GNoME still performs highly competitively with MLIPs trained on the data. Finally, we note that the poor performance of the GNoME potential in Molybdenum is consistent with all other methods and with recent work [11, 8], highlighting challenges of current existing MLIPs on certain elements. Fig.3d in the main text displays the performance of the GNoME potential in comparison to other general-purpose potentials, including a zero-estimator (i.e. predicting a value of 0 for each force component), as well as the M3GNET [9] and CHGNet [10] potentials. Figures 38 - 43 show the performance of the pretrained GNoME potential in comparison to the M3GNET potential trained on the M3GNET data. In addition they also show the performance of the GNoME potential on two subset of the combined test data, namely a) only the melted test structures including a vacancy as well as b) on the 300K bulk data.

Model	N_{train}	Has seen Ni	RMSE, Forces
BPNN	263	Yes	67.3
MTP	263	Yes	26.9
M3GNET, trained on data	263	Yes	37.4
M3GNET, zero-shot	0	Not reported	342.2
CHGNet, zero-shot	0	Not reported	140.6
GNoME, zero-shot	0	No	71.8

Supplementary Table 1: Zero-shot performance on GNoME model in comparison to methods trained on the Nickel data set. RMSE in units of [meV/Å].

Model	N_{train}	Has seen Cu	RMSE, Forces
BPNN	262	Yes	63.0
MTP	262	Yes	13.5
M3GNET, trained on data	262	Yes	17.0
M3GNET, zero-shot	0	Not reported	153.9
CHGNet, zero-shot	0	Not reported	269.2
GNoME, zero-shot	0	Yes	40.0

Supplementary Table 2: Zero-shot performance on GNoME model in comparison to methods trained on the Copper data set. RMSE in units of [meV/Å].

Model	N_{train}	Has seen Li	RMSE, Forces
BPNN	241	Yes	63.4
MTP	241	Yes	13.2
M3GNET, trained on data	241	Yes	22.1
M3GNET, zero-shot	0	Not reported	69.8
CHGNet, zero-shot	0	Not reported	69.7
GNoME, zero-shot	0	Yes	34.9

Supplementary Table 3: Zero-shot performance on GNoME model in comparison to methods trained on the Lithium data set. RMSE in units of [meV/Å].

Model	N_{train}	Has seen Mo	RMSE, Forces
BPNN	194	Yes	198.7
MTP	194	Yes	148.1
M3GNET, trained on data	194	Yes	193.7
M3GNET, zero-shot	0	Not reported	565.4
CHGNet, zero-shot	0	Not reported	529.7
GNoME, zero-shot	0	Yes	272.9

Supplementary Table 4: Zero-shot performance on GNoME model in comparison to methods trained on the Molybdenum data set. RMSE in units of [meV/Å].

Model	N_{train}	Has seen Si	RMSE, Forces
BPNN	214	Yes	174.2
MTP	214	Yes	88.1
M3GNET, trained on data	214	Yes	102.8
M3GNET, zero-shot	0	Not reported	396.7
CHGNet, zero-shot	0	Not reported	228.0
GNoME, zero-shot	0	Yes	128.5

Supplementary Table 5: Zero-shot performance on GNoME model in comparison to methods trained on the Silicon data set. RMSE in units of [meV/Å].

Model	N_{train}	Has seen Ge	RMSE, Forces
BPNN	228	Yes	124.3
MTP	228	Yes	70.3
M3GNET, trained on data	228	Yes	76.4
M3GNET, zero-shot	0	Not reported	507.3
CHGNet, zero-shot	0	Not reported	243.4
GNoME, zero-shot	0	Yes	104.9

Supplementary Table 6: Zero-shot performance on GNoME model in comparison to methods trained on the Germanium data set. RMSE in units of [meV/Å].

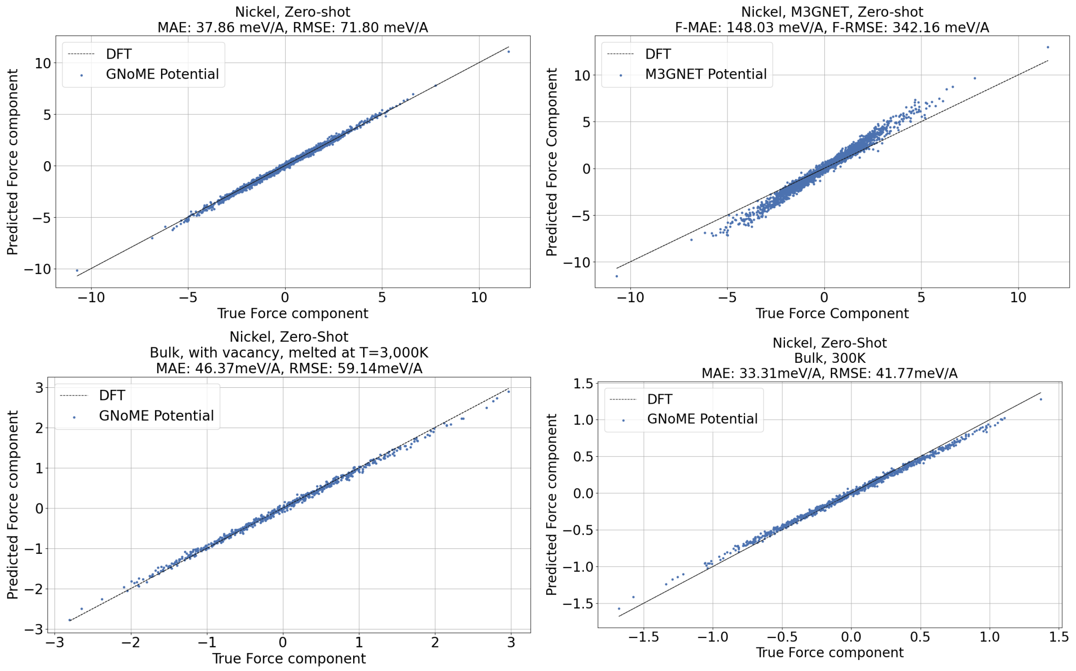

Supplemental Figure 38: **Zero-shot performance on Nickel.** Force components in units of [eV/Å] a) GNoME potential evaluated on all Nickel test set structures. The GNoME potential has never seen pure Nickel and has not been trained on data sampled from MD. In addition, the test data include surfaces, whereas the GNoME potential has only ever seen bulk structures. b) Performance of the M3GNET potential. c) GNoME potential evaluated on Nickel at T=3,000K in the melt, including a vacancy. The GNoME potential has never seen melted structures and has never seen a vacancy. d) GNoME potential evaluated on Nickel at T=300K.

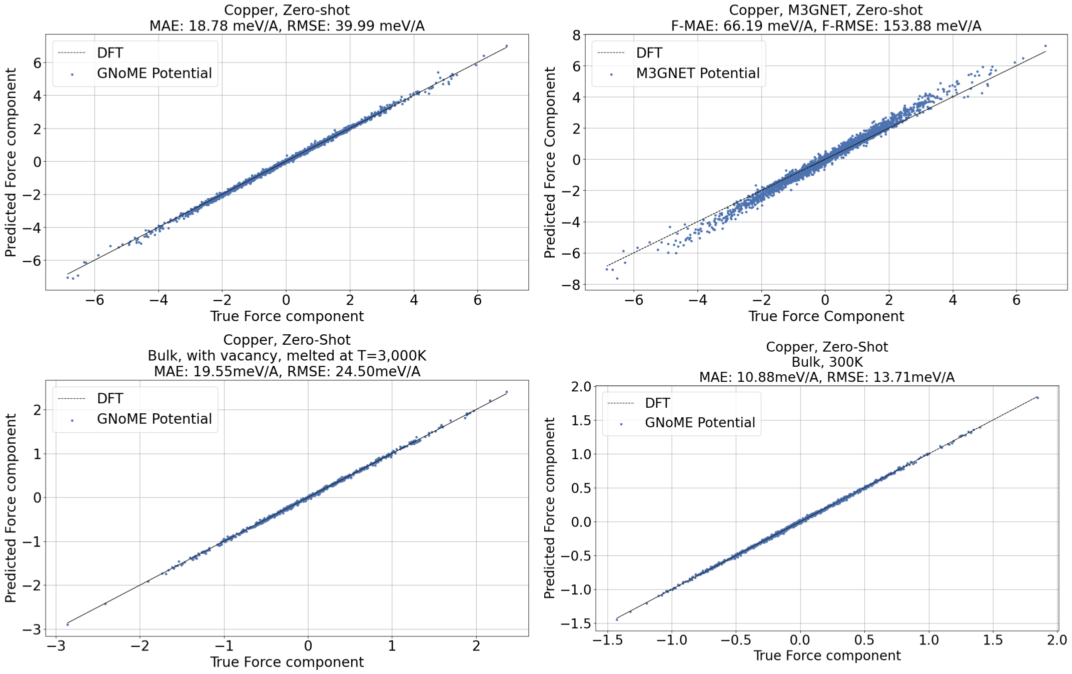

Supplemental Figure 39: **Zero-shot performance on Copper.** Force components in units of [eV/Å] a) GNoME potential evaluated on all Copper test set structures. The GNoME potential has not been trained on data sampled from MD. In addition, the test data include surfaces, whereas the GNoME potential has only ever seen bulk structures. b) Performance of the M3GNET potential. c) GNoME potential evaluated on Copper at T=3,000K in the melt, including a vacancy. The GNoME potential has never seen melted structures and has never seen a vacancy. d) GNoME potential evaluated on Copper at T=300K.

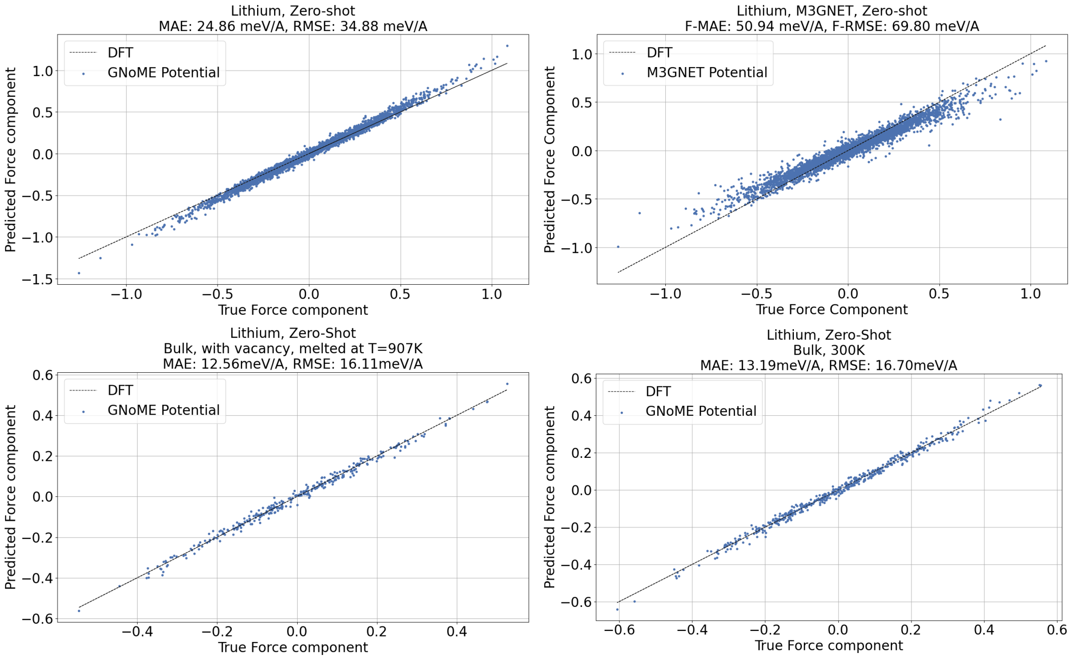

Supplemental Figure 40: **Zero-shot performance on Lithium.** Force components in units of [eV/Å] a) GNoME potential evaluated on all Lithium test set structures. The GNoME potential has not been trained on data sampled from MD. In addition, the test data include surfaces, whereas the GNoME potential has only ever seen bulk structures. b) Performance of the M3GNET potential. c) GNoME potential evaluated on Lithium at T=907K in the melt, including a vacancy. The GNoME potential has never seen melted structures and has never seen a vacancy. d) GNoME potential evaluated on Lithium at T=300K.

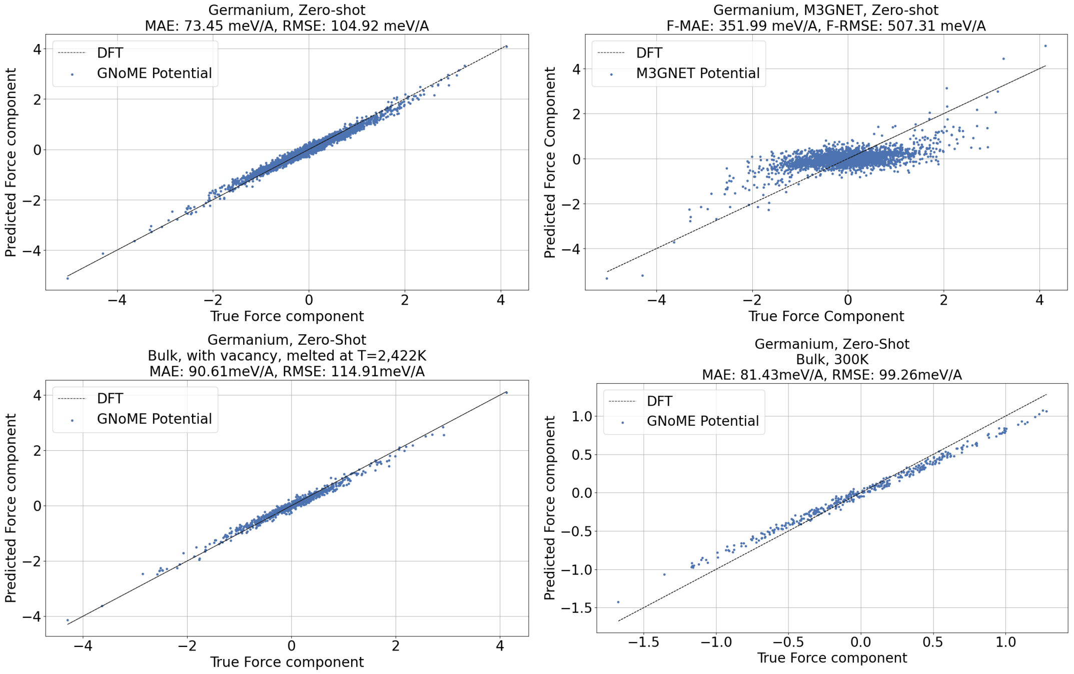

Supplemental Figure 41: **Zero-shot performance on Germanium.** Force components in units of [eV/Å] a) GNoME potential evaluated on all Germanium test set structures. The GNoME potential has not been trained on data sampled from MD. In addition, the test data include surfaces, whereas the GNoME potential has only ever seen bulk structures. b) Performance of the M3GNET potential. c) GNoME potential evaluated on Germanium at T=2,422K in the melt, including a vacancy. The GNoME potential has never seen melted structures and has never seen a vacancy. d) GNoME potential evaluated on Germanium at T=300K.

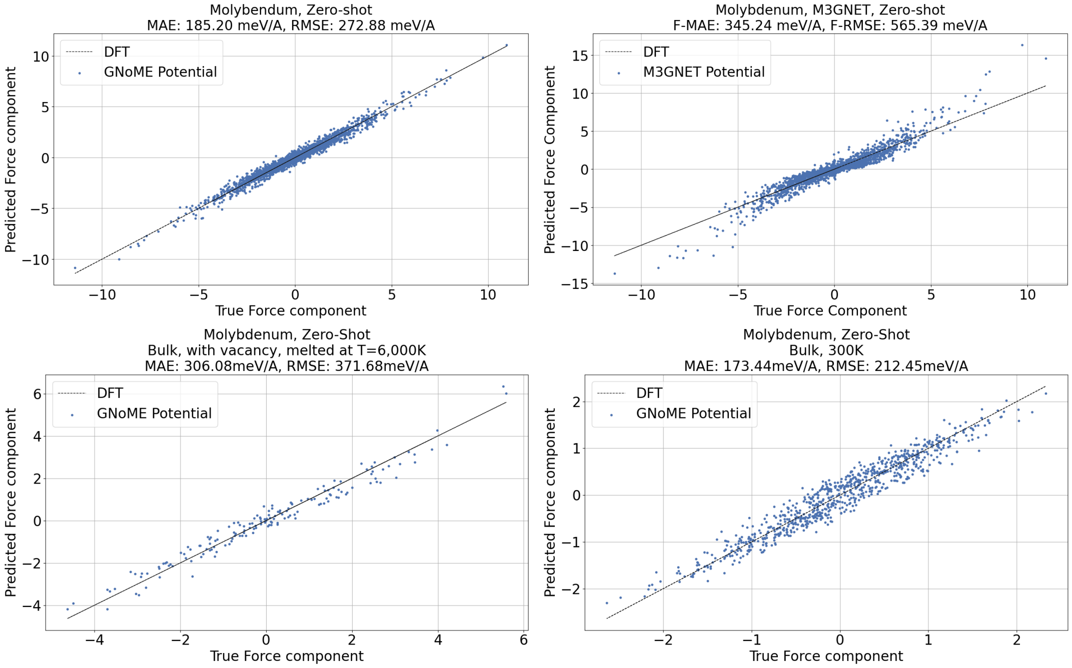

Supplemental Figure 42: **Zero-shot performance on Molybdenum.** Force components in units of [eV/Å] a) GNoME potential evaluated on all Molybdenum test set structures. The GNoME potential has not been trained on data sampled from MD. In addition, the test data include surfaces, whereas the GNoME potential has only ever seen bulk structures. b) Performance of the M3GNET potential. c) GNoME potential evaluated on Molybdenum at T=6,000K in the melt, including a vacancy. The GNoME potential has never seen melted structures and has never seen a vacancy. d) GNoME potential evaluated on Molybdenum at T=300K.

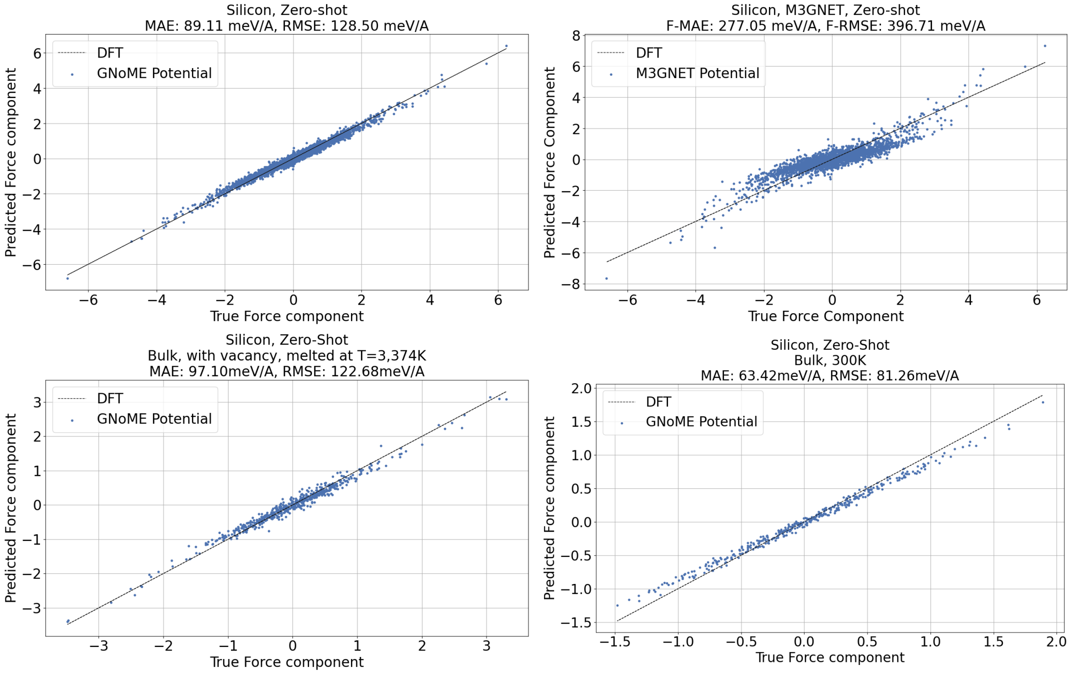

Supplemental Figure 43: **Zero-shot performance on Silicon.** Force components in units of [eV/Å] a) GNoME potential evaluated on all Silicon test set structures. The GNoME potential has not been trained on data sampled from MD. In addition, the test data include surfaces, whereas the GNoME potential has only ever seen bulk structures. b) Performance of the M3GNET potential. c) GNoME potential evaluated on Silicon at T=3,374K in the melt, including a vacancy. The GNoME potential has never seen melted structures and has never seen a vacancy. d) GNoME potential evaluated on Silicon at T=300K.

6.5 Matbench Discovery

With the recent interest in using data-driven approaches to materials discovery, a number of post-hoc analysis methods have been designed to evaluate the performance of the associated machine-learning models in this domain, ranging from fingerprint-based methods to alternate graph neural networks [12]. In particular, we focus on the Matbench Discovery tasks, which have been designed based on the WBM set of structural substitutions. The IS2RE (input structure to relaxed energy) task takes as input the original structural substitution and uses models to predict the relaxed energy. Associated metrics then include the MAE of the relaxed energy as well as precision/recall to determine if the model would have helped improve the efficiency of materials discovery. Best models to date are based on general-purpose interatomic potentials. We apply the pre-trained GNoME models described in this paper to the Matbench Discovery tasks and find state-of-the-art results across all metrics. Results are presented in Table 7, showcasing the improvement from the scale that GNoME brings. We did not find the performance to vary based on whether the WBM structure has a composition that has our training hash or test hash.

While the GNoME data enable a significant improvement on the Matbench Discovery task, the improvement realized from the improved efficiency of our models is more significant on the broader distribution of the materials, as defined by our convex hull (see Fig. 1d). This is because the Matbench Discovery task only includes elemental substitutions, whereas our discoveries include SAPS which lead to a more diverse and subsequently more difficult stability prediction task.

Model	F1 ↑	DAF ↑	Prec ↑	Acc ↑	TPR ↑	TNR ↑	MAE ↓	RMSE ↓	R^2 ↑
GNoME	**0.81**	**4.86**	**0.83**	**0.94**	**0.80**	**0.97**	**0.03**	**0.08**	**0.78**
CHGNet	0.58	3.06	0.52	0.84	0.66	0.88	0.07	0.11	0.61
M3GNet	0.57	2.67	0.45	0.80	0.77	0.81	0.07	0.11	0.60
MACE	0.57	2.78	0.47	0.81	0.72	0.83	0.07	0.11	0.63
ALIGNN	0.56	2.92	0.50	0.83	0.65	0.87	0.09	0.15	0.27
MEGNet	0.51	2.70	0.45	0.81	0.57	0.86	0.13	0.20	-0.28
CGCNN	0.51	2.63	0.45	0.81	0.59	0.85	0.14	0.23	-0.62
CGCNN+P	0.51	2.40	0.41	0.78	0.67	0.80	0.11	0.18	0.03
Wrenformer	0.48	2.13	0.36	0.74	0.69	0.75	0.10	0.18	-0.04
BOWSR	0.44	1.91	0.32	0.68	0.74	0.67	0.12	0.16	0.14
Voronoi RF	0.34	1.51	0.26	0.67	0.51	0.70	0.14	0.21	-0.31
Dummy	0.19	1.00	0.17	0.68	0.23	0.77	0.12	0.18	0.00

Supplementary Table 7: Matbench discovery results using the potential enabled by the GNoME dataset. For results, we relax the input structures, relax for 500 steps, and evaluate the outputted energies. The results showcase that the interatomic potentials trained as part of this work showcase state-of-the-art performance on downstream tasks. Results from all other models other than GNoME were taken from the original Matbench Discovery task. The maximum possible discovery acceleration factor (DAF) is ≈ 6, and arrows are used to indicate improvement for a given metric.

6.6 Model Limitations

The GNoME pretraining dataset is limited to bulk, inorganic crystals. As a result, it is not expected to perform well on systems such as surfaces, clusters, or organic systems. In fact, the pretrained potential performed poorly on surface systems in the elemental datasets, as well as on strained systems. However, these data are still kept in the test data to enable a fair comparison to other methods that were trained explicitly on data from the target distribution. Finetuning from a pretrained checkpoint may still improve performance on a data set containing types of structures not in our pretraining dataset. But we note that the performance of the model will likely be dependent on how similar the pretraining data is to the downstream task. In addition, while we see strong zero-shot performance in many cases, we also observed materials in which the zero-shot performance is lacking. In these cases, fine-tuning can quickly improve performance while exhibiting strongly improved robustness (see robustness results).

Supplementary References

[1] Ceder, G. & Garbulsky, G. Ground state dmgrams for ternary fcc alloys (1993).

[2] Hegde, V. I., Aykol, M., Kirklin, S. & Wolverton, C. The phase stability network of all inorganic materials. *Science advances* **6**, eaay5606 (2020).

[3] Jain, A. *et al.* Commentary: The materials project: A materials genome approach to accelerating materials innovation. *APL materials* **1**, 011002 (2013).

[4] Saal, J. E., Kirklin, S., Aykol, M., Meredig, B. & Wolverton, C. Materials design and discovery with high-throughput density functional theory: the open quantum materials database (oqmd). *Jom* **65**, 1501–1509 (2013).

[5] Fu, X. *et al.* Forces are not enough: Benchmark and critical evaluation for machine learning force fields with molecular simulations. *arXiv preprint arXiv:2210.07237* (2022).

[6] Hestness, J. *et al.* Deep learning scaling is predictable, empirically. *arXiv preprint arXiv:1712.00409* (2017).

[7] Kaplan, J. *et al.* Scaling laws for neural language models. *arXiv preprint arXiv:2001.08361* (2020).

[8] Zuo, Y. *et al.* Performance and cost assessment of machine learning interatomic potentials. *The Journal of Physical Chemistry A* **124**, 731–745 (2020).

[9] Chen, C. & Ong, S. P. A universal graph deep learning interatomic potential for the periodic table. *arXiv preprint arXiv:2202.02450* (2022).

[10] Deng, B. *et al.* Chgnet: Pretrained universal neural network potential for charge-informed atomistic modeling. *arXiv preprint arXiv:2302.14231* (2023).

[11] Owen, C. J. *et al.* Complexity of many-body interactions in transition metals via machine-learned force fields from the tm23 data set. *arXiv preprint arXiv:2302.12993* (2023).

[12] Riebesell, J. *et al.* Matbench discovery–an evaluation framework for machine learning crystal stability prediction. *arXiv preprint arXiv:2308.14920* (2023).
